# ZEB1-mediated biogenesis of circNIPBL sustains the metastasis of bladder cancer via Wnt/β-catenin pathway

**DOI:** 10.1186/s13046-023-02757-3

**Published:** 2023-08-02

**Authors:** Yuanlong Li, Yao Kong, Mingjie An, Yuming Luo, Hanhao Zheng, Yan Lin, Jiancheng Chen, Jin Yang, Libo Liu, Baoming Luo, Jian Huang, Tianxin Lin, Changhao Chen

**Affiliations:** 1 Department of Urology, Sun Yat-sen Memorial Hospital, 107 Yanjiangxi Road, Yuexiu District, Guangzhou, 510120 Guangdong P. R. China; 2 Guangdong Provincial Key Laboratory of Malignant Tumor Epigenetics and Gene Regulation, State Key Laboratory of Oncology in South China, Sun Yat-sen Memorial Hospital, Guangzhou, Guangdong P. R. China; 3 Department of Pancreatic Surgery, Department of General Surgery, Guangdong Provincial People’s Hospital, Guangdong Academy of Medical Sciences, Guangzhou, Guangdong P. R. China; 4 Department of Urology, Affiliated Hospital of Chengdu University, Chengdu, Sichuan P. R. China; 5 Department of Ultrasound, Sun Yat-sen Memorial Hospital, Guangzhou, Guangdong P. R. China

**Keywords:** circRNA biogenesis, Bladder cancer, ZEB1, Wnt signaling pathway, Positive feedback loop

## Abstract

**Background:**

Circular RNAs (circRNAs) circularized by back-splicing of pre-mRNA are widely expressed and affected the proliferation, invasion and metastasis of bladder cancer (BCa). However, the mechanism underlying circRNA biogenesis in mediating the distant metastasis of BCa still unexplored.

**Methods:**

RNA sequencing data between BCa and normal adjacent tissues was applied to identify the differentially expressed circRNAs. The functions of circNIPBL in BCa were investigated via a series of biochemical experiments. The Clinical significance of circNIPBL was examined in a cohort of larger BCa tissues.

**Results:**

In the present study, we identified a novel circRNA (hsa_circ_0001472), circNIPBL, which was significantly upregulated and had great influence on the poor prognosis of patients with BCa. Functionally, circNIPBL promotes BCa metastasis in vitro and in vivo. Mechanistically, circNIPBL upregulate the expression of Wnt5a and activated the Wnt/β-catenin signaling pathway via directly sponged miR-16-2-3p, leading to the upregulation of ZEB1, which triggers the EMT of BCa. Moreover, we revealed that ZEB1 interacted with the flanking introns of exons 2–9 on NIPBL pre-mRNA to trigger circNIPBL biogenesis, thus forming a positive feedback loop. Importantly, circNIPBL overexpression significantly facilitated the distant metastasis of BCa in the orthotopic bladder cancer model, while silencing ZEB1 remarkably blocked the effects of metastasis induced by circNIPBL overexpression.

**Conclusions:**

Our study highlights that circNIPBL-induced Wnt signaling pathway activation triggers ZEB1-mediated circNIPBL biogenesis, which forms a positive feedback loop via the circNIPBL/miR-16-2-3p/Wnt5a/ZEB1 axis, supporting circNIPBL as a novel therapeutic target and potential biomarker for BCa patients.

**Graphical Abstract:**

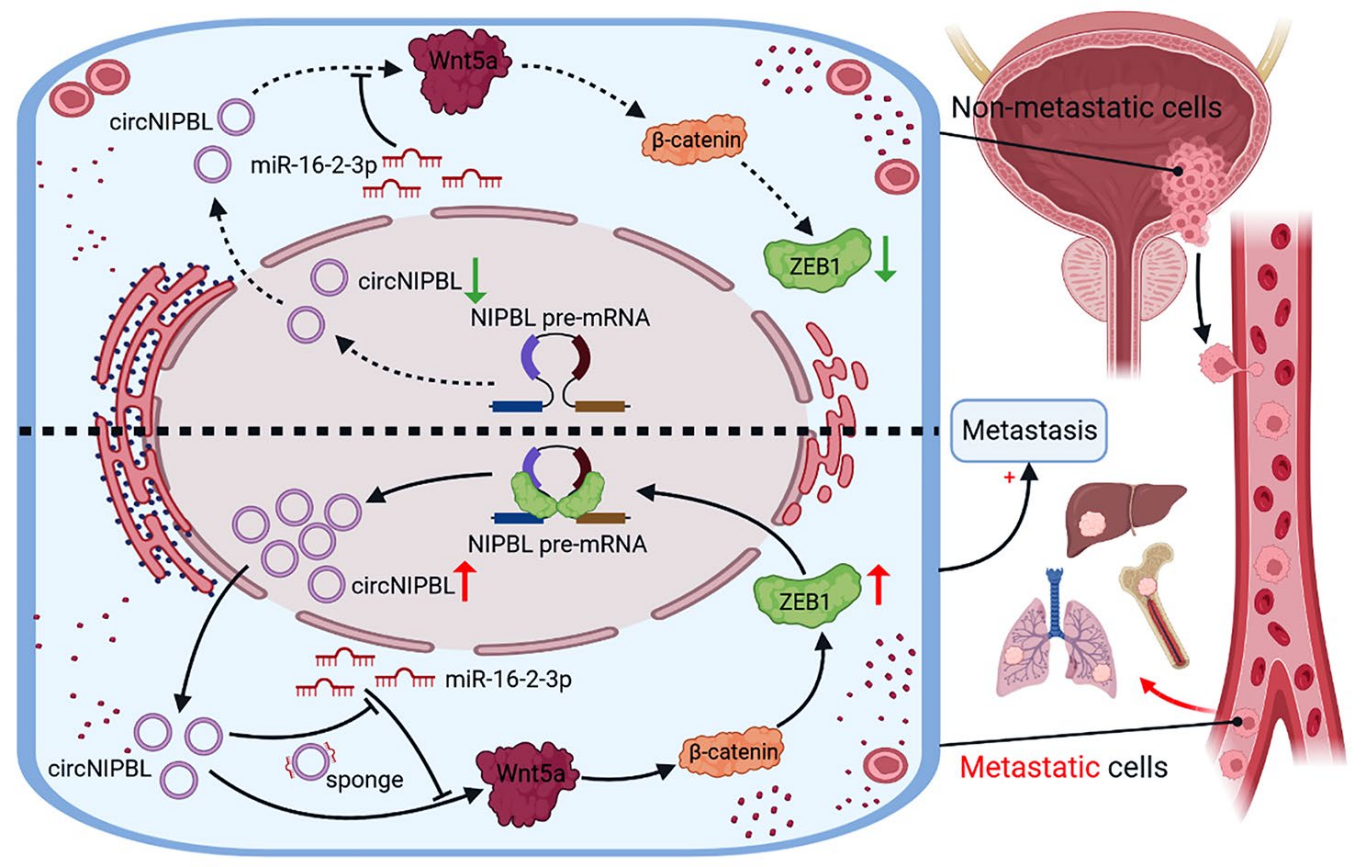

**Supplementary Information:**

The online version contains supplementary material available at 10.1186/s13046-023-02757-3.

## Introduction

Bladder cancer (BCa) is one of the most common urothelial carcinomas of the urinary tract with high morbidity and mortality, accounting for 3.2% of all cancers worldwide and leading to approximately 213,000 deaths per year [1, 2]. Based on the tumor depth, approximately 75% of BCa cases are classified as non muscle-invasive bladder cancer (NMIBC), and 25% are termed muscle-invasive bladder cancer (MIBC) [1, 3]. Due to the distant metastasis at the time of diagnosis, epidemiological evidence suggests that 5-15% of patients with MIBC have poor prognosis and a lower 5-year survival rate [1, 4, 5]. The common clinical treatments for patients with distant metastasis, such as radical cystectomy or chemotherapy, do not completely eradicate the cancer and have severe side effects [6–8]. The dilemma of metastasis patients is caused by the difficulty in detecting metastasis at early stages and the lack of effective targeted therapeutics [6]. Therefore, further investigation into the biological and molecular mechanisms underlying distant metastasis is important to identify new diagnostic and therapeutic targets for BCa.

The Wnt signaling pathway is a canonical signal transduction cascade that functions as a vital regulator of cancer progression, in which Wnt5a, the ligand for members of the frizzled family of seven transmembrane receptors, plays a critical role by regulating the expression of multiple regulatory factors [9–11]. The abnormal expression of Wnt5a sustainably activates the Wnt signaling pathway to regulate the proliferation, differentiation and metastasis of tumor cells [12–14]. Emerging evidence has shown that sustainable activation of the Wnt signaling pathway promotes the progression and metastasis of multiple advanced cancers via EMT (epithelial-mesenchymal transition) and has a significant correlation with poor prognosis [11, 15–17]. In addition, systemically blocking the expression of Wnt5a via inhibitors remarkably inhibited malignant progression and distant metastasis in vivo [18, 19]. Therefore, further investigation of the molecular mechanism and key regulators of the Wnt5a-mediated sustainable activation of the Wnt signaling pathway could provide an effective target for treatment of BCa.

With the covalently closed loop structures, circular RNAs (circRNAs) were defined as a kind of endogenous noncoding RNAs, which have been verified to play a regulatory role in cancer biology [20–23]. In general, circRNAs produced by noncanonical splicing of precursor mRNA, called back-splicing, are mainly expressed at a lower level than their linear transcripts and exert superior biological functions [24–26]. Recent research has showed that the aberrant regulation of the biogenesis of circRNAs plays a functional role in regulating intracellular signal transduction, which leads to the uncontrolled development of a variety of cancers [26, 27]. For example, E2F1 and EIF4A3 sustainably promote the circularization of circSEPT9 to facilitate the migration and invasion of breast cancer [28]. Nevertheless, the mechanisms and functions underlying the circRNAs biogenesis in distance metastasis of BCa still remain unknown.

In this study, we identified a circRNA, circNIPBL (hsa_circ_0001472), that was significantly upregulated and had great influence on the poor prognosis of patients with BCa. Overexpressing circNIPBL promoted the distance metastasis of BCa in vitro and in vivo. Mechanistically, circNIPBL activated the Wnt5a/β-catenin signaling pathway to upregulate ZEB1 expression by sponging miR-16-2-3p. Moreover, ZEB1 bound to the flanking intron 1^45999–46005nt^ and intron 9^618–624nt^ of NIPBL pre-mRNA and facilitated its back-splicing to promote the biogenesis of circNIPBL, which formed a positive feedback loop and promoted the metastasis of BCa. Our study reveals the underlying mechanism of ZEB1-mediated circNIPBL biogenesis to form a ZEB1/circNIPBL/Wnt5a positive feedback loop in regulating BCa metastasis, indicating that circNIPBL might be an attractive therapeutic target and biomarker in BCa.

## Results

### circNIPBL is positively associated with bladder cancer metastasis

**Fig. 1:**
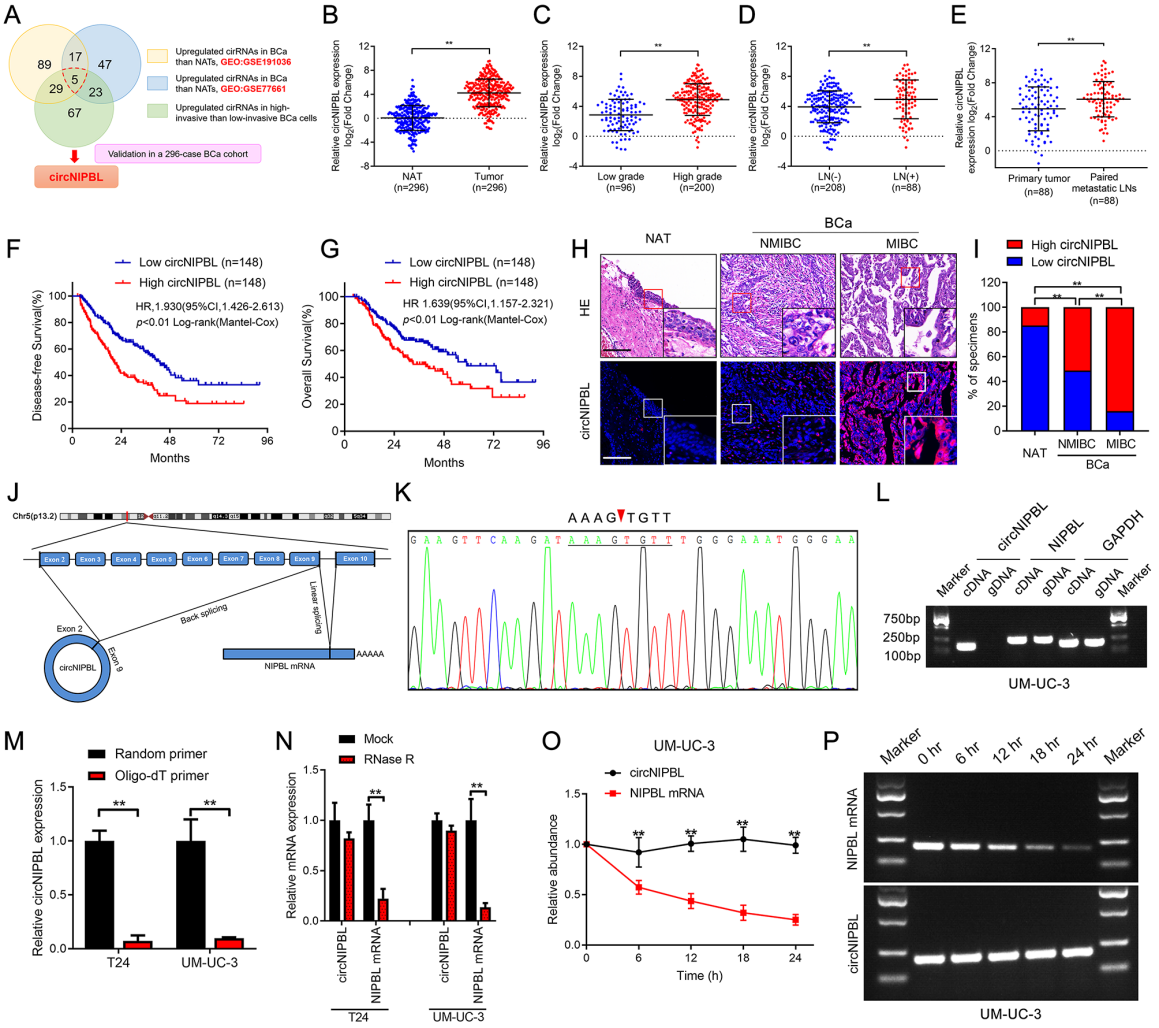
The identification and characterization of circNIPBL in BCa. (**A**) Schematic illustration for screening the upregulated circRNAs in bladder cancer tissues and high-invasive bladder cancer cells. (**B**) qRT-PCR for the circNIPBL expression in BCa tissues (n = 296) paired with NATs (n = 296). (**C, D**) qRT-PCR analysis of the circNIPBL expression in 296 BCa tissues with respect to pathological grade (C) and LN status (D). (**E**) qRT-PCR for the circNIPBL expression in primary tumor (n = 88) paired with metastatic LNs (n = 88). (**F, G**) Kaplan-Meier survival curves for OS (F) and DFS (G) of low and high circNIPBL expression level in patients with BCa. The median expression level of circNIPBL was taken as the cutoff value. (**H, I**) Representative images (H) and proportion (I) for RNA fluorescence in situ hybridization showed that circNIPBL was upregulated in MIBC tissues compared with NMIBC tissues. Scale bar = 50 μm and 20 μm. (**J**) Schematic illustration showing the genomic loci of the NIPBL gene and the circNIPBL derived from exon 2 to 9 of NIPBL. (**K**) Sanger sequencing for the back-splice junction site of circNIPBL. (**L**) PCR analysis for circNIPBL and NIPBL in the cDNA and gDNA of UM-UC-3 cells. GAPDH was used as normal control. (**M**) qRT-PCR analysis of circNIPBL expression using random primers or oligo-dT primers. (**N**) circNIPBL and NIPBL expression in BCa cells analyzed by qRT-PCR following RNase R treatment in UM-UC-3 and T24 cells. (**O, P**) Actinomycin D assay (O) and agarose gel electrophoresis assay (P) to assess the stability of circNIPBL and NIPBL mRNA in UM-UC-3 cells at the indicated time points. The statistical difference was assessed through the nonparametric Mann–Whitney *U* test in B-G and the *χ*^*2*^ test in I; and the two-tailed Student *t* test in M-O. Error bars show the SD from three independent experiments. *p < 0.05 and **p < 0.01

To explore the crucial circRNAs that contribute to BCa metastasis, we performed high-throughput sequencing of BCa specimens and paired normal adjacent tissues (NATs) from 4 patients (GSE191036) and 2 paired high/low-invasive BCa cell lines established previously were performed to explore the crucial circRNAs that contribute to BCa metastasis [29]. Next, we intersected our sequencing data with a public RNA sequencing data (GSE77661) to identify 5 upregulated circRNAs in BCa tissues compared with NATs (fold change ≥ 2.0 and p < 0.05) (Fig. 1A). Then, these circRNAs from the above screening were further evaluated in a larger cohort of 296 BCa patients to reveal that circNIPBL (hsa_circ_0001472), which was the most significantly upregulated one in BCa tissues (Fig. 1B). Then, we evaluated the affection of circNIPBL expression in the clinicopathology of BCa. The results revealed that patients with high-grade and lymphatic metastasis had higher expression levels of circNIPBL than those with low-grade and non-lymphatic metastasis (Fig. 1C and D). Furthermore, the expression of circNIPBL was higher in metastatic LN than corresponding primary tumors (Fig. 1E). Kaplan‒Meier survival analyses showed that circNIPBL overexpression was correlated with shorter overall survival (OS) and disease-free survival (DFS) of BCa patients (Fig. 1F and G). Cox univariate and multivariate analyses showed that circNIPBL expression was an independent predictor of poor prognosis in BCa patients (Table 1, S1, S2). Moreover, RNA fluorescence in situ hybridization (FISH) analysis showed that circNIPBL had higher expression levels in BCa than in NAT (Fig. 1H and I). Taken together, the above results suggest that circNIPBL is markedly upregulated and affected the poor survival of BCa patients.

**Table 1:** Correlation between circNIPBL expression and clinicopathologic characteristics of BCa patients (n = 296)

Characteristics	No. of cases	circNIPBL expression		
		Low	High	*P*-value^i^
**Total cases**	296	148	148	
**Sex**				0.608
Male	210	103	107	
Female	86	45	41	
**Age**				0.635
< 65	118	57	61	
≥ 65	178	91	87	
**Grade**				**0.001** ^**^
Low	96	74	22	
High	200	74	126	
**T stage**				**0.001** ^**^
T1	54	43	11	
T2	79	55	24	
T3	121	48	73	
T4	42	12	30	
**Lymphatic metastasis**				**0.001** ^**^
Negative	208	112	89	
Positive	88	29	59	

Abbreviations: No. of cases = number of cases; T grade = tumor grade. ^i^Chi-square test, * *p* < 0.05, ** *p* < 0.01

Since circRNAs have different secondary structures from their counterpart genes, we first investigated the circular characteristics of circNIPBL, which was generated from exons 2–9 of the NIPBL gene [24, 30]. Sanger sequencing validated the back-splicing structure of circNIPBL (Fig. 1J K). PCR analysis was performed to amplify circNIPBL circular transcripts and NIPBL linear transcripts. The results showed that NIPBL existed in both complementary DNA (cDNA) and genomic DNA (gDNA), while circNIPBL was only detected in cDNA, indicating that circNIPBL was formed by head-to-tail splicing rather than genomic rearrangements (Fig. 1L and Fig. S1A). In addition, the reverse transcription product from the random primer contained a higher expression level of circNIPBL than that of the oligo-dT primer, suggesting the less of a poly-A tail in circNIPBL (Fig. 1M). Then, we evaluated the stability of circNIPBL. After treatment with RNase R, NIPBL expression was obviously decreased, while circNIPBL was not affected (Fig. 1N). An actinomycin D assay showed that circNIPBL had a longer half-life than NIPBL mRNA, indicating the stable structure of circNIPBL (Fig. 1O and P, Fig. S1B and S1C). Taken together, the above results proved that circNIPBL contains a stable covalently closed loop structure derived from exons 2–9 of the NIPBL gene locus.

### circNIPBL enhances the migration and invasion of BCa in vitro

**Fig. 2:**
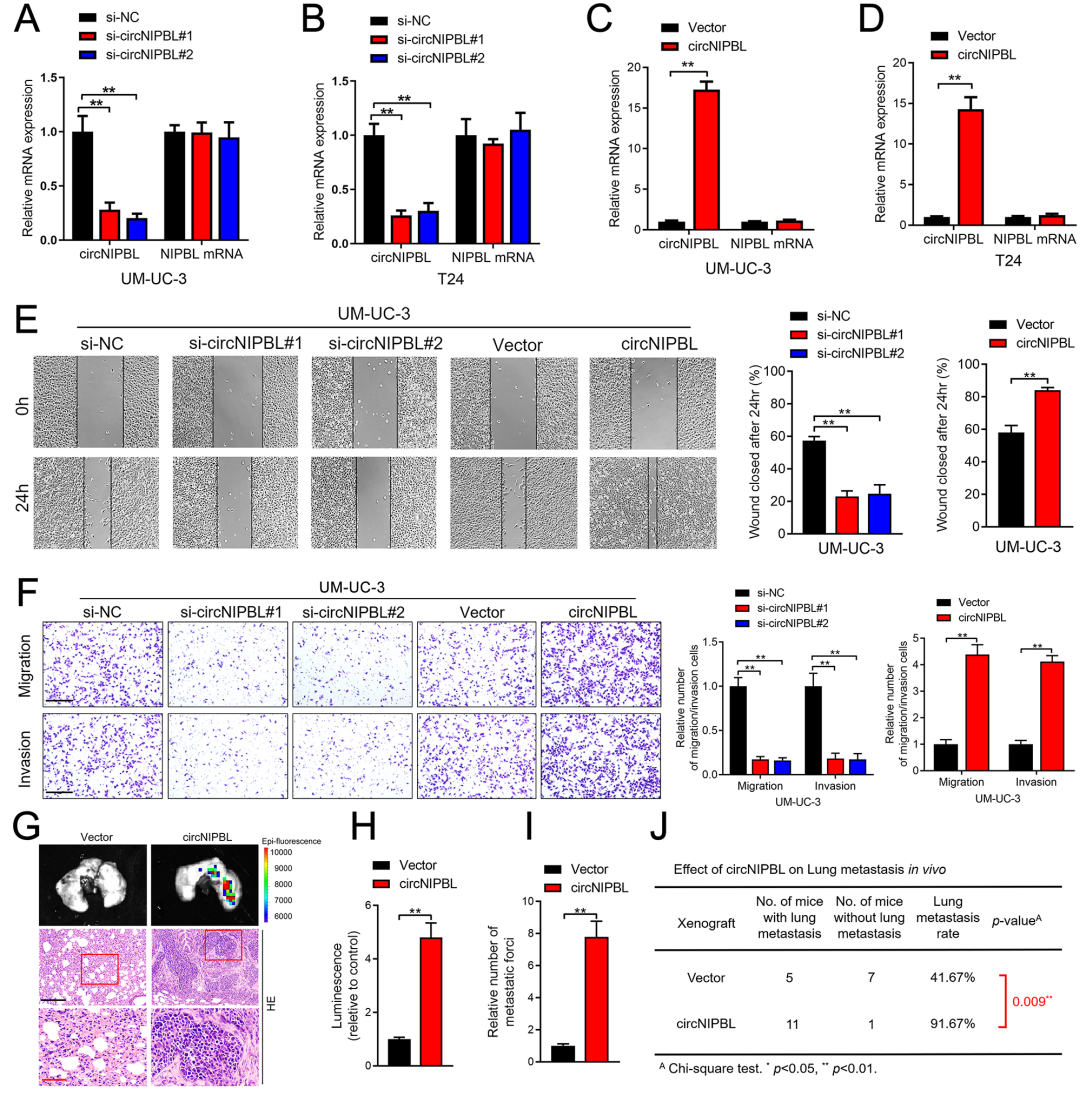
circNIPBL promotes the proliferation, migration, and invasion of BCa cells in vitro and in vivo. (**A–D**) qRT-PCR was used to assess the expression level of circNIPBL and NIPBL in circNIPBL knockdown (A, B), circNIPBL overexpression (C, D), and paired control BCa cells. (**E**) Representative images and quantification of Wound healing assay of UM-UC-3 cells treated with circNIPBL-downregulated or -overexpressing. Scale bar = 100 μm. (**F**) Representative images and quantification of Transwell migration and Matrigel invasion assays of UM-UC-3 cells treated with circNIPBL-downregulated or -overexpressing. Scale bar = 100 μm. (**G-I**) Representative bioluminescence images (G) and quantification (H, I) of in tail-vein injection model. Scale bar = 50 μm and 20 μm. (**J**) The ratio of lung metastasis was calculated for all groups (n = 12 per group). The statistical difference was assessed with one-way ANOVA followed by Dunnett tests in A, B, E and F; and the two-tailed Student *t* test in C, D, E, F, H and I; and the *χ*^*2*^ test in J. Error bars show the SD from three independent experiments. *p < 0.05 and **p < 0.01. H&E, hematoxylin and eosin

To assess the function of circNIPBL in BCa cells, we further explored whether circNIPBL promotes metastasis in vitro. First, qRT‒PCR analysis showed that circNIPBL was overexpressed in UM-UC-3 and T24 cells instead of in RT112 and 5637 cells, which are less invasive, and SV-HUC cells, which are normal bladder epithelial cells (Fig. S1D). Then, the results of qRT‒PCR analysis validated that circNIPBL was successfully upregulated or downregulated after ectopic expression or knockdown of circNIPBL in T24 and UM-UC-3 cells through transfection with circNIPBL-overexpressing plasmid and small interfering RNAs (siRNAs) (Fig. 2A and D). Wound healing assays showed that the migration of BCa cells was facilitated after overexpressing circNIPBL, while silencing circNIPBL significantly suppressed the migration of BCa cells (Fig. 2E and Fig. S1E). Likewise, Transwell assays demonstrated that the overexpression of circNIPBL enhanced the invasion and migration ability of BCa cells. After silencing circNIPBL, the migration and invasion ability of BCa cells was obviously reduced (Fig. 2F and Fig. S1F). These results indicate that circNIPBL overexpression enhances the migration and invasion ability of BCa cells.

### CircNIPBL promotes the metastasis of BCa in vivo

Considering the affection of circNIPBL in BCa cells, the regulatory role of circNIPBL in BCa metastasis was further evaluated *in vivo.* To build a tail vein injection mouse model, BCa cells were transfected with luciferase-labeled circNIPBL overexpression plasmid and injected into the tail vein of nude mice. The fluorescence intensity detected by in vivo imaging system (*IVIS*) in the circNIPBL-overexpressing group was obviously increased rather than the control group, indicating that circNIPBL facilitates the lung metastasis of luciferase-labeled BCa cells (Fig. 2G H). Then, we evaluated the number of lung metastatic foci in mice, which showed that circNIPBL overexpression significantly promoted lung metastasis of BCa cells (Fig. 2I). The IHC assay suggested that compared with the control group, the incidence of lung metastasis was higher in the circNIPBL-overexpressing group (Fig. 2J). The above results demonstrate that circNIPBL promotes the metastasis of BCa in vivo.

### CircNIPBL directly binds with miR-16-2-3p

**Fig. 3:**
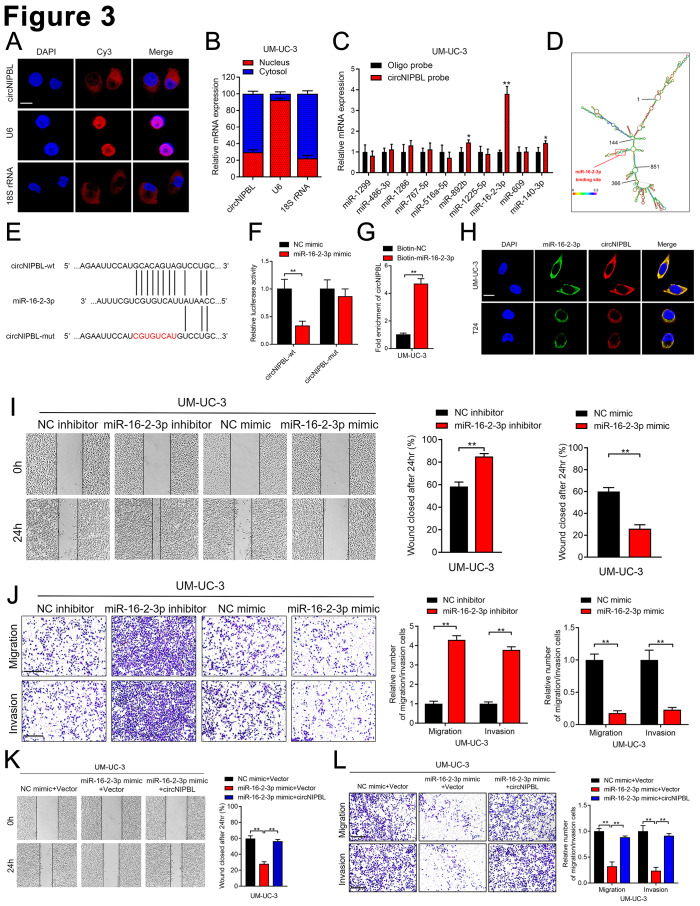
circNIPBL serves as a sponge for miR-16-2-3p in BCa cells. (**A**) FISH assay was used to detect the cellular localization of circNIPBL. Scale bar = 5 μm. (**B**) Subcellular fractionation assay was used to confirm the cellular localization of circNIPBL in UM-UC-3 cells. U6 was used for the nuclear control and 18 S rRNA was used for the cytoplasmic control. (**C**) The expression level of ten predicted target miRNAs of circNIPBL were analyzed by qRT-PCR in UM-UC-3 cells. (**D**) RNAalifold was used to predict the secondary structure of circNIPBL. (**E**) Schematic illustrating the sequence alignment of circNIPBL with miR-16-2-3p. (**F**) Dual luciferase reporter assays showed that the luciferase activities of the circNIPBL-wt plasmid or circNIPBL-mut plasmid quantified following co-transfection with either the miR-16-2-3p or control mimics. (**G**) qRT-PCR analysis showed the circNIPBL captured by biotinylated miR-16-2-3p. (**H**) The co-localization of circNIPBL and miR-16-2-3p was detected by FISH assay. Scale bar = 5 μm. (**I**) Representative images and quantification of Wound healing assay showed the migration capability of UM-UC-3 cells transfected with miR-16-2-3p mimics or inhibitors. Scale bar = 100 μm. (**J**) Representative images and quantification of Transwell migration and Matrigel invasion assays showed the effect of UM-UC-3 cells transfected with miR-16-2-3p mimics or inhibitors. Scale bar = 100 μm. (**K, L**) Representative images and quantification of wound healing assay (K), Transwell migration and Matrigel invasion assays (L) by indicated UM-UC-3 cells. Scale bar = 100 μm. The statistical difference was assessed with one-way ANOVA followed by Dunnett tests in K and L; and the two-tailed Student *t* test in C, F, G, I and J; and the *χ*^*2*^ test in B. Error bars show the SD from three independent experiments. *p < 0.05 and **p < 0.01

We next investigated the underlying mechanism of circNIPBL in promoting the metastasis of BCa. Since the localization of circRNAs is crucial for their function, we revealed that circNIPBL was basically existed in the cytoplasm of BCa cells by nuclear-cytoplasmic fractionation assays and fluorescence in situ hybridization (FISH) [31, 32] (Fig. 3A and B and Fig. S2A). Given that cytoplasm-localized circRNA primarily functions as competitive endogenous RNA (ceRNA), we predicted 10 candidate miRNAs potentially binding with circNIPBL by CircInteractome and circMir2.0 [33, 34] (Fig. S2B). Then, the probes targeting circNIPBL were used in RNA pull-down assay, which revealed that only miR-16-2-3p was enriched by circNIPBL in BCa cells (Fig. 3C and Fig. S2C). Then, we analyzed the secondary structure of circNIPBL and found a potential sequence complementary to miR-16-2-3p (Fig. 3D and E). The RNA pull-down assay with biotinylated miR-16-2-3p probes and dual-luciferase reporter assay verified that circNIPBL directly interacts with miR-16-2-3p (Fig. 3F and G and Fig. S2D). Similarly, FISH assays demonstrated that circNIPBL and miR-16-2-3p co-localized in the cytoplasm of BCa cells (Fig. 3H). Together, the above results show that circNIPBL directly binds with miR-16-2-3p.

Next, we further investigated whether circNIPBL served as a sponge of miR-16-2-3p to promotes the metastasis of BCa. Wound healing assays and transwell assays indicated that miR-16-2-3p overexpression inhibited the migration and invasion of BCa cells, while miR-16-2-3p knockdown showed the opposite effect (Fig. 3I J and Fig. S2E-S2H). Moreover, circNIPBL overexpression significantly attenuated the suppression of miR-16-2-3p on the migration and invasion of BCa cells (Fig. 3K L, Fig. S2I and S2J). Collectively, these data suggest that circNIPBL promote the migration and invasion of BCa cells by via acting as a sponge of miR-16-2-3p.

### circNIPBL overexpression upregulates Wnt5a by targeting miR-16-2-3p

**Fig. 4:**
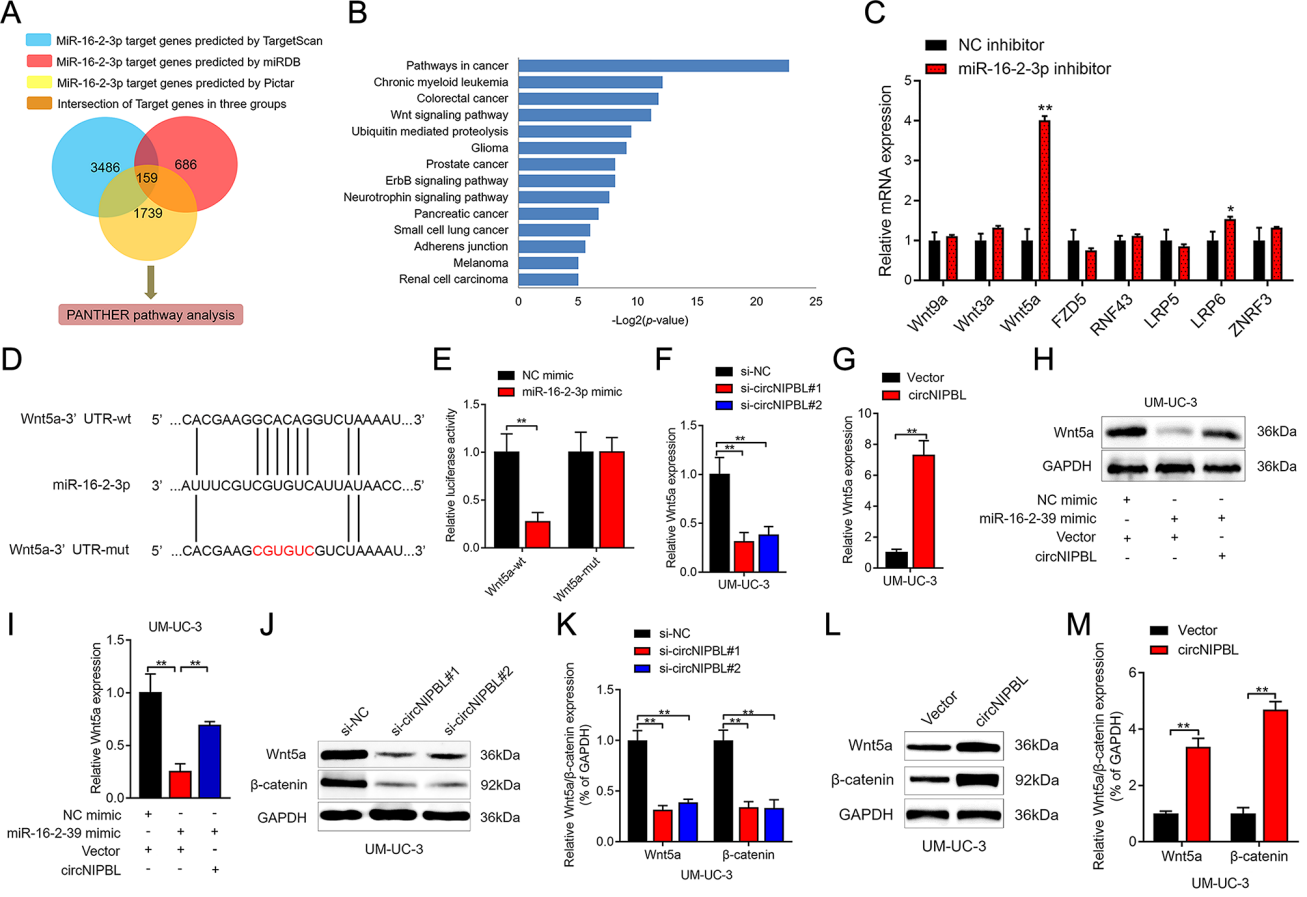
circNIPBL attenuates miR-16-2-3p-mediated Wnt signaling suppression. (**A**) Schematic illustration showing the downstream targets of miR-16-2-3p as predicted by the TargetScan, Pictar and miRDB databases. (**B**) KEGG pathway enrichment analysis with PANTHER database of BCa. (**C**) qRT-PCR analysis of Wnt signaling pathway-related genes in circNIPBL overexpressing BCa cells. (**D**) Schematic illustration showed the alignment of miR-16-2-3p with Wnt5a and the mutagenesis nucleotides were indicated by the red portion. (**E**) The luciferase activities of the Wnt5a-wt plasmid or Wnt5a-mut plasmid quantified following transfecting control mimic or miR-16-2-3p mimic into BCa cells. (**F, G**) qRT-PCR analysis of the impact of circNIPBL knockdown (F) or circNIPBL overexpression (G) on Wnt5a expression in UM-UC-3 cells. (**H, I**) Representative images (H) and quantification (I) of Western blotting assay of Wnt5a in indicated UM-UC-3 cells. (**J-M**) Western blotting assay showed the expression levels of Wnt5a and β-catenin after circNIPBL knockdown (J, K) or circNIPBL overexpression (L, M) in UM-UC-3 cells. The statistical difference was assessed with one-way ANOVA followed by Dunnett tests in F, I and K; and the two-tailed Student *t* test in C, E, G and M. Error bars show the SD from three independent experiments. *p < 0.05 and **p < 0.01

TargetScan, Pictar and miRDB were applied to identify the downstream target genes of miR-16-2-3p. After intersecting the predicted genes from the above databases, 159 genes were shown to be potential downstream target genes of miR-16-2-3p (Fig. 4A). Subsequently, the PANTHER database was used in pathway analysis to predict 159 downstream target genes, and the Wnt signaling pathway was the most significantly enriched pathway among all enriched pathways (Fig. 4B). Next, we investigated the expression level of crucial genes in the Wnt signaling pathway after knocking down miR-16-2-3p in BCa cells. The results revealed that Wnt5a was significantly increased while inhibiting miR-16-2-3p expression, indicating that Wnt5a was the downstream target of miR-16-2-3p (Fig. 4C).

MiRNAs bind to the 3’UTR and suppress the expression of target genes [35, 36]. To verify that Wnt5a was the downstream gene of miR-16-2-3p, the sequence of miR-16-2-3p and the 3’UTR of Wnt5a were analyzed and the results showed that Wnt5a 3’UTR harbored complementary sequences to miR-16-2-3p (Fig. 4D). Then, we mutated the complementary sequence in the Wnt5a 3’UTR and constructed luciferase plasmids. The luciferase intensity of Wnt5a-wt was reduced in group transfected with miR-16-2-3p mimics, while no obvious change was found in the group transfected with Wnt5a-mut in the dual luciferase assay (Fig. 4E and Fig. S3A). Then, we investigated whether circNIPBL can regulate the expression of Wnt5a via miR-16-2-3p. The Wnt5a expression was reduced after circNIPBL downregulated and increased while circNIPBL expression was upregulated (Fig. 4F and G, Fig. S3B and S3C). Furthermore, Western blotting assays revealed that the miR-16-2-3p mimic inhibited the expression of Wnt5a in BCa cells, while overexpressing circNIPBL significantly attenuated the suppression of miR-16-2-3p (Fig. 4H and I, Fig. S3D and S3E). Taken together, these results indicated that circNIPBL acts as a miR-16-2-3p sponge to activate the Wnt5a expression.

### CircNIPBL upregulated the expression of ZEB1 by activating the wnt signaling pathway in BCa

Previous researches have revealed that Wnt5a mediates biological processes via activation of the canonical Wnt pathway [37, 38]. To verify whether circNIPBL promoted BCa metastasis depending on the Wnt/β-catenin pathway, we overexpressed circNIPBL in UM-UC-3 and T24 cells and detected the expression level of core regulators in the pathway by Western blotting assays, in which the results suggested that the expression of Wnt5a and β-catenin was upregulated. Additionally, downregulating circNIPBL obviously inhibited Wnt5a and β-catenin expression in BCa cells, suggesting that circNIPBL upregulated Wnt5a expression to activate the Wnt/β-catenin pathway in BCa (Fig. 4J M and Fig. S3F-S3I).

**Fig. 5:**
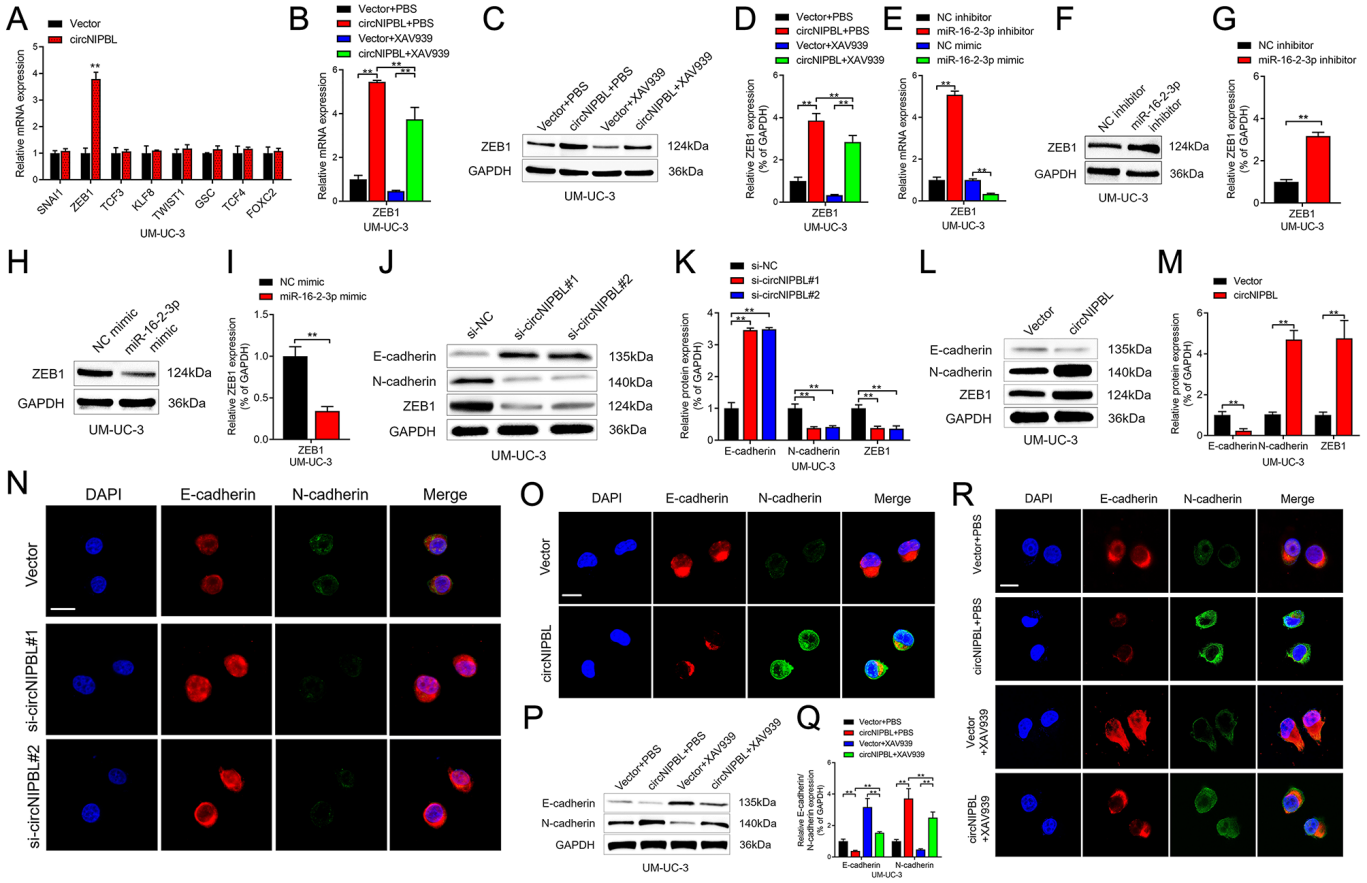
circNIPBL facilitates the expression level of ZEB1. (**A**) qRT-PCR analysis showed that the expressions of downstream targets of Wnt signaling pathway in circNIPBL overexpressing BCa cells. (**B**) qRT-PCR analysis of ZEB1 expression in indicated UM-UC-3 cells. (**C, D**) Representative images (C) and quantification (D) of Western blotting assay of ZEB1 in indicated UM-UC-3 cells. (**E**) qRT-PCR analysis of ZEB1 expression in indicated UM-UC-3 cells. (**F-I**) Representative images and quantification of Western blotting assay of ZEB1 in UM-UC-3 cells transfected with miR-16-2-3p inhibitors (F, G) and mimics (H, I). (**J-M**) Representative images and quantification of Western blotting assay of N-cadherin, E-cadherin, ZEB1 after circNIPBL knockdown (J, K) or circNIPBL overexpression (L, M) in UM-UC-3 cells. (**N, O**) The expression of N-cadherin and E-cadherin was detected by IF assay in circNIPBL knockdown (N) or circNIPBL overexpression (O) UM-UC-3 cells. Scale bar = 5 μm. (**P, Q**) Representative images (P) and quantification (Q) of Western blotting assay of N-cadherin and E-cadherin in indicated UM-UC-3 cells. (**R**) The expression of N-cadherin and E-cadherin was detected by IF assay in indicated UM-UC-3 cells. Scale bar = 5 μm. The statistical difference was assessed with one-way ANOVA followed by Dunnett tests in B, D, E, K and Q; and the two-tailed Student *t* test in A, G, I and M. Error bars show the SD from three independent experiments. *p < 0.05 and **p < 0.01

The Wnt signaling pathway is known to correlate with the pathological grade, EMT and poor prognosis of BCa [39–41]. Hence, we investigated the expression of key regulators of EMT through qRT‒PCR assays in BCa cells, and the results showed that ZEB1 was upregulated after overexpressing circNIPBL (Fig. 5A and Fig. S4A). Notably, after applying XAV939, an inhibitor of the Wnt pathway, to BCa cells, the upregulation of ZEB1 induced by circNIPBL overexpression was reversed. (Figure 5B and D and Fig. S4B-S4D). In addition, the results of Western blot assays and qRT‒PCR showed that the protein and mRNA levels of ZEB1 was negatively associated with mirR-16-2-3p, suggesting that circNIPBL upregulates ZEB1 via sponging mirR-16-2-3p and activating Wnt signaling pathway. (Figure 5E and I and Fig. S4E-S4I). Furthermore, Western blot assays and immunofluorescence (IF) assays confirmed that circNIPBL upregulated the expression of the mesenchymal marker N-cadherin and suppressed the expression of the epithelial marker E-cadherin, while downregulating circNIPBL had the opposite effect (Fig. 5J and O and Fig. S4J-S4O). In addition, the upregulation of N-cadherin and the downregulation of E-cadherin mediated by circNIPBL were reversed by XAV939 treatment in BCa cells, suggesting that circNIPBL promotes EMT via the Wnt signaling pathway (Fig. 5P and R and Fig. S4P-S4R). Together, these results revealed that circNIPBL upregulates the expression of ZEB1 and induces EMT in BCa by activating the Wnt signaling pathway.

## ZEB1 binds to the flanking introns of NIPBL pre-mRNA to trigger circNIPBL biogenesis

**Fig. 6:**
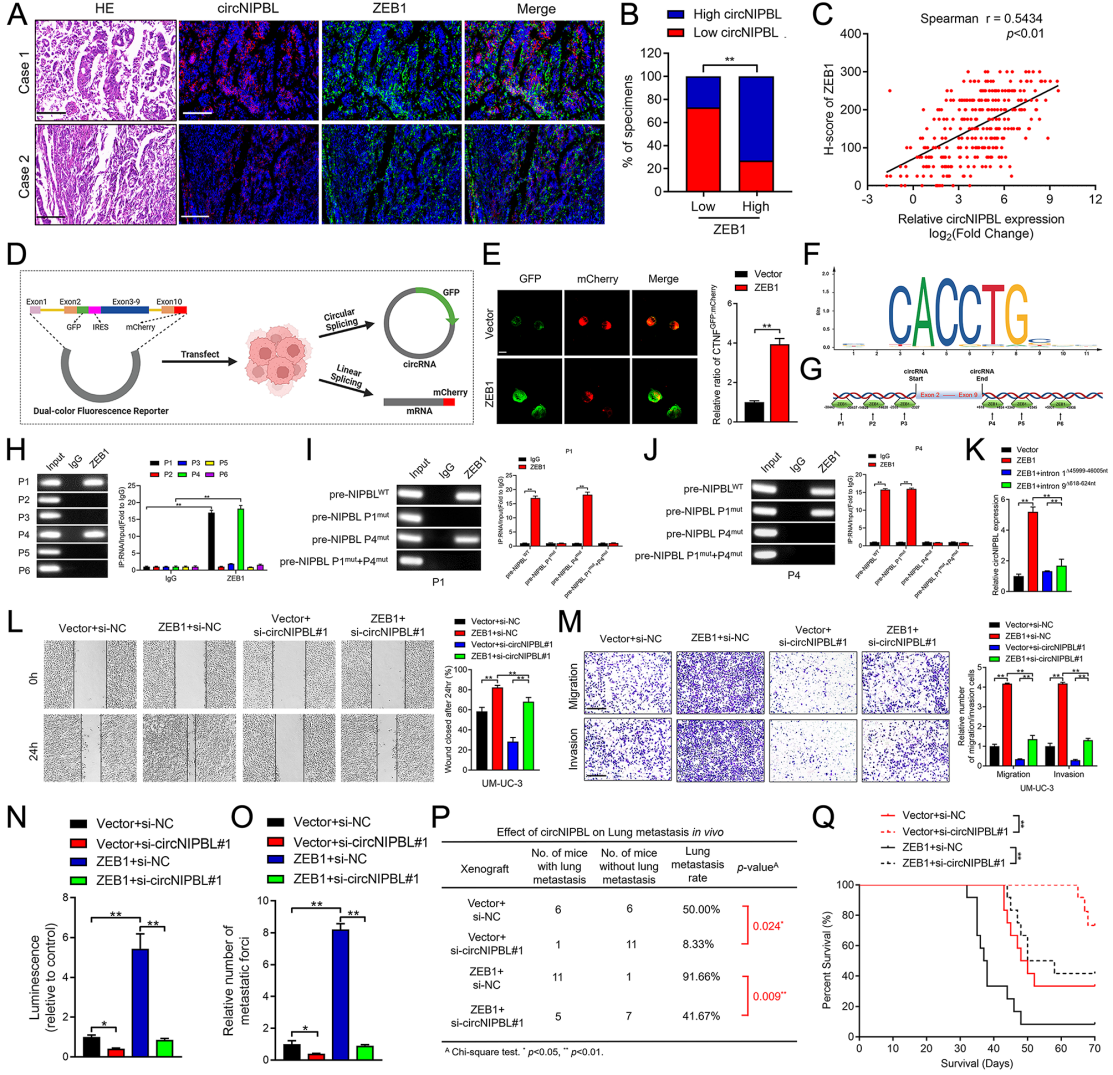
ZEB1 binds to the flanking intron of pre-mRNA to trigger circNIPBL biogenesis. (**A-C**) Representative images (A) and quantification (B, C) of ZEB1–indicated circNIPBL expression was detected by RNA fluorescence in situ hybridization assay in BCa tissues. Scale bar = 50 μm. (**D**) Schematic illustration of the dual-color fluorescence reporter. (**E**) Representative images and quantification of circNIPBL and NIPBL mRNA expression in BCa cells. Scale bar = 5 μm. (**F**) The potential ZEB1 binding motifs in NIPBL pre-mRNA were predicted by JASPAR online database. (**G**) ZEB1-binding sites in the flanking regions of NIPBL pre-mRNA were predicted by Circinteractome online database. (**H**) Representative images and quantification of RIP assay using anti-ZEB1 antibody to validate the putative binding sites. (**I, J**) Representative images and quantification of RIP assay using anti-ZEB1 antibody to validate the indicated binding sites P1 (I) and P4 (J) after mutation. (**K**) qRT-PCR analysis showed that the expression of circNIPBL after deleting the binding sites in flanking introns. (**L, M**) Representative images and quantification of Wound healing (L) and Transwell migration and Matrigel invasion (M) assays in indicated UM-UC-3 cells. Scale bar = 100 μm. (**N, O**) Quantification of the luminescence (N) and metastasis forci (O) in tail-vein injection model. (**P**) The ratio of lung metastasis was calculated for all groups (n = 12 per group). (**Q**) The survival time of the ZEB1-transduced tumor bearing mice. The statistical difference was assessed through the nonparametric Mann–Whitney *U* test in B, Q and the *χ*^*2*^ test in C, P; and one-way ANOVA followed by Dunnett tests in H, K, L, M, N and O; and the two-tailed Student *t* test in E, I and J. Error bars show the SD from three independent experiments. *p < 0.05 and **p < 0.01

Recent studies have shown that circRNA produced by pre-mRNA back-splicing plays an essential role in cancer progression [26, 42, 43]. Therefore, we further clarified whether ZEB1 could affect the biogenesis of circNIPBL by a positive feedback loop. qRT‒PCR assays revealed that circNIPBL was increased or reduced after upregulating or downregulating ZEB1 in BCa cells, respectively, while the NIPBL pre-mRNA expression remained invariant (Fig. S5A-S5H). Moreover, the ratio of circNIPBL and NIPBL mRNA was increased in ZEB1-overexpressing BCa cells, indicating that ZEB1 facilitated the biogenesis of circNIPBL instead of parental gene expression (Fig. S5I-S5L). Furthermore, immunohistochemistry and RNA FISH analysis in a larger cohort of clinical specimens revealed that the expression of ZEB1 positively correlated with circNIPBL, which indicated that ZEB1 promoted the biogenesis of circNIPBL (Fig. 6A C). To provide a detected tool, a dual-color fluorescence reporter was constructed to simultaneously quantify the expression of both linear and circRNA splicing [43]. The reporter was designed such that circNIPBL biogenesis gives rise to IRES-mediated translation of GFP and the linear NIPBL pre-mRNA produced from the reporter undergoes mCherry expression (Fig. 6D). The GFP–mCherry ratio was markedly increased after overexpressing ZEB1 in BCa cells, suggesting that the expression level of circNIPBL was upregulated (Fig. 6E).

Previous studies showed that the biogenesis of circular RNAs was regulated by RNA binding proteins (RBPs) via directly binding to the flanking introns of splicing exons and induced the circularization of circRNAs [26, 43]. Then, we predicted the binding motif of ZEB1 in the JASPAR online database and revealed the potential binding sites in the flanking introns of exons 2–9 of NIPBL pre-mRNA by bioinformatic analysis (Fig. 6F and G). RNA immunoprecipitation (RIP) assays using anti-ZEB1 verified that ZEB1 significantly enriched the flanking introns of NIPBL pre-mRNA compared with the IgG group (Fig. 6H). Next, mutation of the flanking intron 1^45999–46005nt^ and intron 9^618–624nt^ of NIPBL pre-mRNA binding sites markedly decreased the flanking intron enrichment by ZEB1, which indicated that ZEB1 binds NIPBL pre-mRNA in intron 1^45999–46005nt^ and intron 9^618–624nt^ (Fig. 6I J). Moreover, the upregulation of circNIPBL was reversed by deleting the binding sites of flanking introns in ZEB1-overexpressing cells (Fig. 6K). These results indicated that ZEB1 binds to the flanking region on NIPBL pre-mRNA to promote the back-splicing of circNIPBL.

## ZEB1-mediated circNIPBL biogenesis sustains wnt signaling activation in BCa by forming a positive feedback loop

The formation of a positive feedback loop triggering the metastasis signaling cascade plays a crucial role in cancer progression [44, 45]. Given that ZEB1 was essential to circNIPBL biogenesis, we further examined whether circNIPBL formed a positive feedback loop via the Wnt5a/ZEB1 axis. Overexpression of ZEB1 promoted tumor metastasis in BCa cells, and knocking down the expression of circNIPBL significantly reduced ZEB1-induced metastasis, as shown by the wound healing assay (Fig. 6L and Fig. S5M). In addition, the promotion of BCa cell migration and invasion induced by ZEB1 overexpression was reversed when circNIPBL expression was knocked down, indicating that ZEB1-mediated circNIPBL biogenesis promotes BCa distant metastasis in vitro (Fig. 6M and Fig. S5N). To explore whether the circNIPBL-mediated Wnt5a/ZEB1 positive feedback loop was involved in the distant metastasis of BCa in vivo, we established a tail vein injection model. IVIS showed that silencing circNIPBL rescued the ZEB1-mediated increase in fluorescence intensity of lung metastatic foci in nude mice (Fig. 6N). Conversely, knocking down circNIPBL markedly abrogated the ZEB1 overexpression-enhanced lung metastatic foci number (Fig. 6O). Moreover, the metastasis rate was markedly raised after overexpressing ZEB1, while downregulating circNIPBL significantly reversed the promotion of BCa metastasis in vivo (Fig. 6P). In addition, we found that knocking down circNIPBL markedly rescued the inhibition of survival time in ZEB1-transduced tumor-bearing mice (Fig. 6Q). Together, these data further supported that the circNIPBL-mediated miR-16-2-3p/Wnt5a/ZEB1 positive feedback loop sustains the activation of EMT and promotes the metastasis of BCa.

### Clinical relevance of the circNIPBL-mediated positive feedback loop in BCa patients

**Fig. 7:**
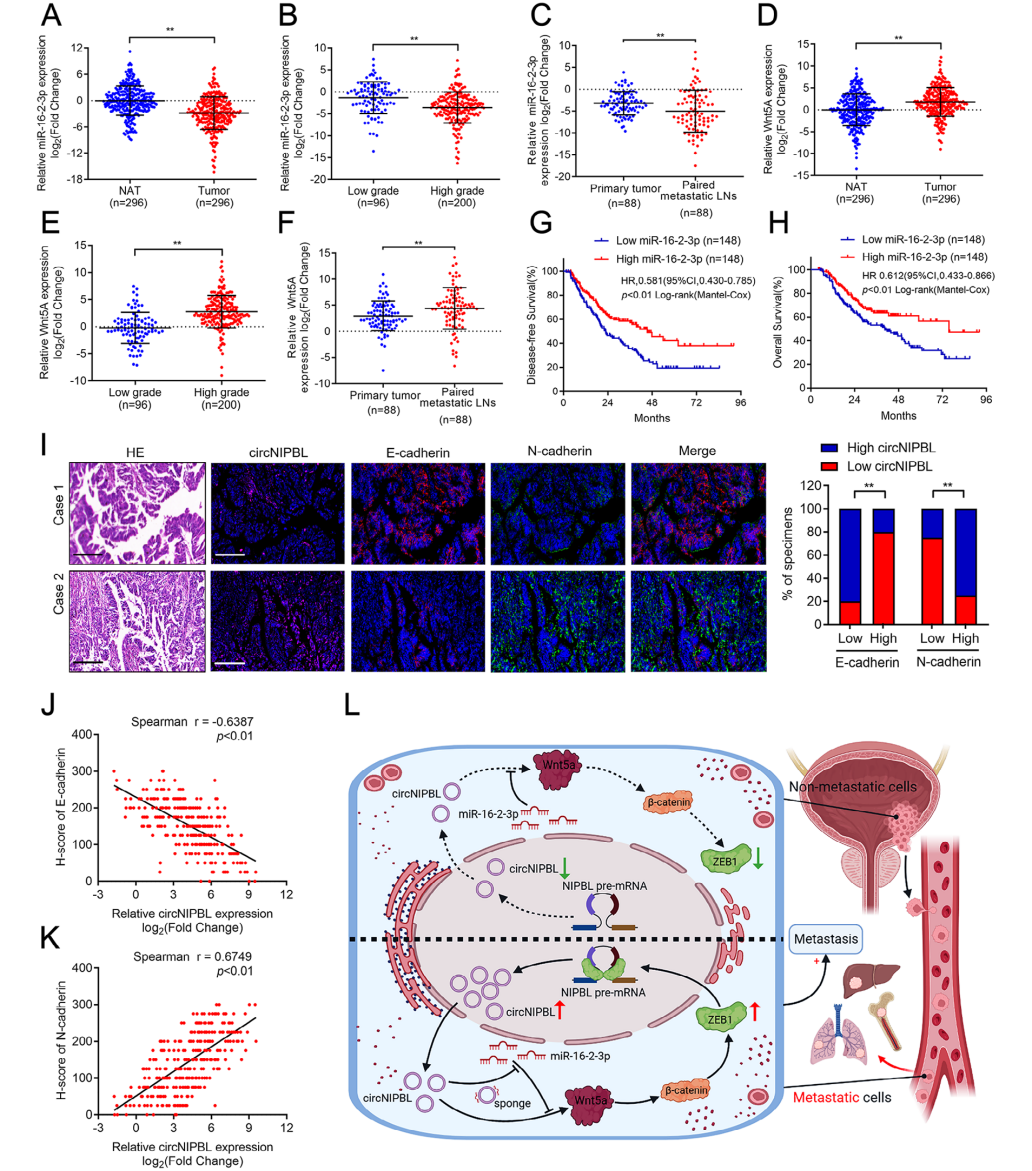
Clinical relevance of the positive feedback loop in patients with BCa. (**A**) qRT-PCR for the miR-16-2-3p expression in BCa tissues (n = 296) paired with NATs (n = 296). (**B**) qRT-PCR analysis showed miR-16-2-3p level in a 296-case BCa tissues with pathological grade. (**C**) qRT-PCR for the miR-16-2-3p expression in primary tumor (n = 88) paired with metastatic LNs (n = 88). (**D**) qRT-PCR for the Wnt5a expression in BCa tissues (n = 296) paired with NATs (n = 296). (**E**) qRT-PCR analysis showed Wnt5a level in a 296-case BCa tissues with pathological grade. (**F**) qRT-PCR for the Wnt5a expression in primary tumor (n = 88) paired with metastatic LNs (n = 88). (**G, H**) Kaplan-Meier survival curves for OS (E) and DFS (F) of low and high miR-16-2-3p expression level in patients with BCa. The median expression level of miR-16-2-3p was taken as the cutoff value. (**I**) Representative images and quantification of circNIPBL–indicated N-cadherin and E-cadherin expression was detected by RNA fluorescence in situ hybridization assay in BCa tissues. Scale bar = 50 μm. (**J, K**) Correlation analysis of circNIPBL and E-cadherin (H), N-cadherin (I) expression in bladder cancer tissues (n = 296). (**L**) Schematic illustrating the potential mechanism of circNIPBL sustains bladder cancer metastasis via a positive feedback loop. The statistical difference was assessed through the nonparametric Mann–Whitney *U* test in A-H, J, K and the *χ*^*2*^ test in I. Error bars show the SD from three independent experiments. *p < 0.05 and **p < 0.01

To investigate the clinical significance of miR-16-2-3p and Wnt5a in BCa patients, qRT‒PCR analysis showed that the expression of miR-16-2-3p was downregulated in a cohort of 296 BCa tissues (Fig. 7A). Moreover, the expression of miR-16-2-3p was negatively correlated with the pathological grade and was lower in metastatic LN than corresponding primary tumors in the large clinical cohort of BCa patients, indicating its vital roles in BCa (Fig. 7B C). In addition, the overexpression of Wnt5a was positively correlated with the pathological status of BCa (Fig. 7D and E). Notably, Wnt5a was significantly upregulated in metastatic LN compared with corresponding primary tumors (Fig. 7F). Kaplan–Meier analysis revealed that BCa patients with miR-16-2-3p overexpression had longer OS and DFS compared with the lower group, which was consistent with the TCGA database (Fig. 7G H). RNA FISH analysis results revealed that patients with circNIPBL overexpression also had a high expression level of N-cadherin and low E-cadherin expression (Fig. 7I). Furthermore, correlation analysis proved that N-cadherin was positively associated with circNIPBL expression, while E-cadherin was negatively associated with circNIPBL expression in 296 BCa tissues (Fig. 7J K). In conclusion, our results demonstrate that circNIPBL-mediated miR-16-2-3p/Wnt5a positive feedback loop promotes the distance metastasis of BCa (Fig. 7L).

## Discussion

Distance metastasis is the major cause of reduced life expectancy and quality of life in patients with BCa, especially in MIBC [3, 4, 46]. Approximately 50% of MIBC patients have poor prognosis, high rate of distant metastases and short survival [47]. Unfortunately, due to the lack of therapeutic targets and effective therapeutic strategies, common therapeutic interventions for distant metastasis are ineffectual [48, 49]. Therefore, investigating the crucial regulatory factors and molecular mechanisms regulating distant metastasis is of great significance for the management of BCa. Herein, we found that circNIPBL was significantly overexpressed in BCa tissues and positively associated with metastasis in BCa patients. Functionally, the upregulation of cicrNIPBL facilitated BCa cell invasion and metastasis in vitro. Moreover, upregulating circNIPBL significantly promoted the distant metastasis of BCa in the tail vein injection mouse model. Our study illustrates that circNIPBL regulates the proliferation and metastasis of BCa cells, indicating that circNIPBL may have potential to act as a therapeutic target for distant metastasis in BCa.

The Wnt signaling pathway is the key cascade regulating carcinogenesis and distant metastasis in multiple cancer types and plays a critical role in EMT by activating the expression of regulatory factors [10, 11, 38]. However, the mechanism underlying hyperactivation of the Wnt signaling pathway in BCa still unclear. In the present study, we revealed that sustainable activation of the Wnt signaling pathway led to the upregulation of ZEB1 in BCa cells, which facilitated EMT and formed a positive feedback loop by promoting the biogenesis of circNIPBL. In addition, using XAV939 to inhibit the Wnt signaling pathway eliminated the upregulation of ZEB1 expression mediated by circNIPBL overexpression. Therefore, our study highlighted a novel mechanism in which circRNA induced sustainable Wnt signaling activation via a ZEB1/circNIPBL/Wnt5a positive feedback loop in BCa, suggesting the essential function of Wnt signaling in regulating the metastasis of BCa.

CircRNAs are produced by the aberrant splicing of pre-mRNA transcripts and are involved in regulating multiple cancer-related biological processes, including growth, metastasis and apoptosis, and have great potential as therapeutic targets and clinical biomarkers for cancers [30, 50–52]. However, the precise mechanism of circRNAs biogenesis in regulating the metastasis of BCa still unclear. In this study, we identified a novel circRNA, circNIPBL, that activated the Wnt/β-catenin signaling pathway by directly sponging miR-16-2-3p and upregulate the ZEB1expression, a transcription factor mainly located in the nucleus. Moreover, we revealed that ZEB1 binds to the flanking region on intron 1 and intron 9 of NIPBL pre-mRNA, which facilitate the biogenesis of circNIPBL and trigger BCa metastasis. These findings highlight a mechanism of ZEB1-mediated circRNA biogenesis in regulating the metastasis of BCa, which supports circNIPBL as a new therapeutic target for BCa patients.

With their special circular covalently bonded structure, circRNAs have a higher stability than linear RNAs [34]. Due to their conservation and tissue specificity, circRNAs are considered to function as special molecular biomarkers for the diagnosis of cancers [53, 54]. Recent studies have proven that circITCH is overexpressed in non-small cell lung cancer tissue and supplements traditional markers to increase the positive diagnosis rate [55]. Similarly, circRNAs are potential diagnostic and prognostic biomarkers in hepatocellular carcinoma [56, 57]. However, the potential of circRNAs as diagnostic biomarkers in distant metastasis of BCa is still elusive. In the present study, we revealed that circNIPBL, which was markedly upregulated in BCa tissues, triggered the uncontrolled progression of BCa and negatively correlated with the survival rate. Moreover, Cox univariate and multivariate analyses revealed that circNIPBL is an independent predictor of poor prognosis in BCa patients. Our findings elucidate the potential of circNIPBL as a biomarker of BCa, providing new insight to solve the problem of traditional biomarkers’ low organ specificity.

In summary, our study uncovered a novel mechanism underlying circNIPBL serving as a sponge of miR-16-2-3p to activate the Wnt signaling pathway, which upregulated the expression of ZEB1 and facilitated the back-splicing of NIPBL mRNA to form a positive feedback loop. Our research provides a new understanding of the significant role of ZEB1-mediated circNIPBL biogenesis and the unique mechanism underlying the ZEB1/circNIPBL/Wnt5a positive feedback loop in the distant metastasis of BCa, suggesting that circNIPBL is an innovative therapeutic target for BCa patients.

## Materials and methods

### Patients and clinical samples

In the present study, a total of 296 BCa specimens and paired NATs were obtained from patients who underwent surgery at Sun Yat-sen Memorial Hospital, Sun Yat-sen University. Two independent professional pathologists diagnosed every urothelial carcinoma sample. All samples were promptly frozen in liquid nitrogen and storaged at -80 °C for long-term preservation until further investigation. All clinical data and the specimens involved in this study were approved by the patients with written informed consent and the approval of the Ethics Committee from Sun Yat-sen Memorial Hospital, Sun Yat-sen University.

### Cell lines and cell culture

The human bladder cancer cell lines T24, UM-UC-3 and 5637, and human normal bladder epithelial cell line (SV-HUC-1) were purchased from American Type Culture Collection (Manassas, VA, USA). The UM-UC-3 cells were cultured in Dulbecco’s modified Eagle’s medium (Gibco, USA). The T24 cells were cultured in Roswell Park Memorial Institute (RPMI) 1640 medium (Gibco, USA), while SV-HUC-1 cells were cultured in Ham’s F12K medium (Gibco, USA). All mediums were supplemented with 10% FBS (HyClone, Israel). All cell lines were cultured in 37 ℃ humid atmosphere containing 5% CO_2_.

### Lentivirus infection and cell transfection

For lentivirus infection, 1 ml of lentivirus working solution was transfected in each well of the 6-well plates seeded with BCa cells and incubated for 8 h. Then, 10 µg/mL puromycin was added to screen the successfully transfected cells after culturing for 72 h.

For cellular transfection, BCa cells were planted in a 6-well plate, and then circNIPBL siRNAs (GenePharma, Shanghai, China), miRNA mimics, and miRNA inhibitor (Sangon Biotech, Shanghai, China) were transfected into these cells with Lipofectamine 3000 or p3000 (Life Technologies, CA, USA) according to the manufacturer’s instructions after one day. The efficiency of transfection was determined by qRT‒PCR or Western blotting analysis.

### Caudal vein metastasis model

Nude mice (four- to five-week-old female) were purchased and fed at the Experimental Animal Center, Sun Yat-sen University (Guangzhou, China) and divided into 2 groups. Next, 5 × 10^5^ luciferase-labeled BCa cells were slowly injected into the caudal vein of each nude mice. After 4 weeks, the mice were imaged under In Vivo Imaging Systems (IVIS) (Xenogen Corporation, Alameda, CA, USA) to evaluate lung metastasis.

### RNA pull-down assay

Block streptomycin-coated magnetic beads with 10 mg/mL bovine serum albumin and yeast tRNA for 3 h at 4 °C on a rotator. Then, biotinylated circNIPBL probes were added and incubated for 2 h at 25 °C. 1 × 10^7^ BCa cells were lysed in 200 µl lysis buffer and the supernatant collected after centrifugating10 min at 120,000 g. After mixed with the beads containing the indicated probes for incubation overnight, the target gene expression was detected by qRT‒PCR analysis.

### Dual-color fluorescence reporter assay

BCa cells (1 × 10^5^) were planted in a 6-well plate, and the plasmids designed and constructed by IGE Biotechnology (Guangzhou, China) were transfected into the cells. After 48 h incubation, the transfected cells were seeded into confocal dishes the day before use. The images were captured via confocal fluorescence microscopy (Carl Zeiss AG, Jenna, Germany).

### Statistical analysis

All quantitative data are examined as the mean and average standard deviation (SD) of at least three independent experiments. The statistical differences of Nonparametric variables were analyzed by χ2 test and parametric variables were analyzed by Student’s *t*-test (2-tailed) and 1-way analysis of variance (ANOVA). H-score was conducted to assess the statistical significance of FISH analysis. Kaplan‒Meier analysis were performed to assess OS and DFS. The multivariate Cox regression model was used to calculate the 95% confidence interval for the independent prognostic factors. *P* < 0.05 was considered statistically significant.

## Electronic supplementary material

Below is the link to the electronic supplementary material.

Supplementary Material 1

Supplementary Material 2

## Data Availability

Data generated or analysed during this study are included in this published article and its supplementary information files and are available from the institutional repository or the corresponding author upon request.

## Abbreviations

BCa: Bladder cancer
NMIBC: Nonmuscle-invasive bladder cancer
MIBC: Muscle-invasive bladder cancer
EMT: Epithelial-mesenchymal transition
circRNA: Circular RNA
NATs: Normal adjacent tissues
OS: Overall survival
DFS: Disease-free survival
FISH: Fluorescence in situ hybridization
cDNA: Complementary DNA
gDNA: Genomic DNA
qRT-PCR: Quantitative real-time PCR
siRNAs: Small interfering RNAs
IVIS: In Vivo Imaging System
ceRNA: Competitive endogenous RNA
IF: Immunofluorescence
RBP: RNA binding protein
RIP: RNA immunoprecipitation
SV-HUC-1: Human normal bladder epithelial cells
DMEM: Dulbecco’s Modified Eagle Medium
RPMI: Roswell Park Memorial Institute
FBS: Fetal bovine serum
SD: Standard deviations
ANOVA: 1-way analysis of variance
χ2 test: Chi-square test
DAPI: 4′, 6-diamidino-2-phenylindole
PBS: Phosphate buffered saline
bp: Base pair
KO: Knockout
WT: Wildtype
TCGA: The Cancer Genome Atlas;

## References

[1] Sanli O, Dobruch J, Knowles MA, et al. Bladder cancer. Nat Rev Dis Primers. 2017;3:17022. 28406148 10.1038/nrdp.2017.22

[2] Wang G, McKenney JK. Urinary bladder Pathology: World Health Organization classification and american Joint Committee on Cancer staging update. Arch Pathol Lab Med. 2019;143(5):571–7. 30044124 10.5858/arpa.2017-0539-RA

[3] Smith AB, Deal AM, Woods ME, et al. Muscle-invasive bladder cancer: evaluating treatment and survival in the National Cancer Data Base. BJU Int. 2014;114(5):719–26. 24325202 10.1111/bju.12601

[4] Jacobs BL, Lee CT, Montie JE. Bladder cancer in 2010: how far have we come? CA Cancer J Clin. 2010;60(4):244–72. 20566675 10.3322/caac.20077

[5] Burger M, Catto JW, Dalbagni G, et al. Epidemiology and risk factors of urothelial bladder cancer. Eur Urol. 2013;63(2):234–41. 22877502 10.1016/j.eururo.2012.07.033

[6] Abufaraj M, Gust K, Moschini M, et al. Management of muscle invasive, locally advanced and metastatic urothelial carcinoma of the bladder: a literature review with emphasis on the role of surgery. Transl Androl Urol. 2016;5(5):735–44. 27785430 10.21037/tau.2016.08.23 PMC5071186

[7] Biot C, Rentsch CA, Gsponer JR, et al. Preexisting BCG-specific T cells improve intravesical immunotherapy for bladder cancer. Sci Transl Med. 2012;4(137):137ra172. 10.1126/scitranslmed.3003586 22674550

[8] Stein JP, Lieskovsky G, Cote R, et al. Radical cystectomy in the treatment of invasive bladder cancer: long-term results in 1,054 patients. J Clin Oncol. 2001;19(3):666–75. 11157016 10.1200/JCO.2001.19.3.666

[9] Chehrazi-Raffle A, Dorff TB, Pal SK, Lyou Y. Wnt/beta-Catenin signaling and Immunotherapy Resistance: Lessons for the treatment of Urothelial Carcinoma. Cancers (Basel). 2021;13(4). 10.3390/cancers13040889 PMC7924395 33672668

[10] Parsons MJ, Tammela T, Dow LE. WNT as a driver and dependency in Cancer. Cancer Discov. 2021;11(10):2413–29. 34518209 10.1158/2159-8290.CD-21-0190 PMC8487948

[11] Zhan T, Rindtorff N, Boutros M. Wnt signaling in cancer. Oncogene. 2017;36(11):1461–73. 27617575 10.1038/onc.2016.304 PMC5357762

[12] Kikuchi A, Yamamoto H, Sato A, Matsumoto S. Wnt5a: its signalling, functions and implication in diseases. Acta Physiol (Oxf). 2012;204(1):17–33. 21518267 10.1111/j.1748-1716.2011.02294.x

[13] Kremenevskaja N, von Wasielewski R, Rao AS, Schofl C, Andersson T, Brabant G. Wnt-5a has tumor suppressor activity in thyroid carcinoma. Oncogene. 2005;24(13):2144–54. 15735754 10.1038/sj.onc.1208370

[14] Nishita M, Enomoto M, Yamagata K, Minami Y. Cell/tissue-tropic functions of Wnt5a signaling in normal and cancer cells. Trends Cell Biol. 2010;20(6):346–54. 20359892 10.1016/j.tcb.2010.03.001

[15] Mittal V. Epithelial mesenchymal transition in Tumor Metastasis. Annu Rev Pathol. 2018;13:395–412. 29414248 10.1146/annurev-pathol-020117-043854

[16] Singh M, Yelle N, Venugopal C, Singh SK. EMT: mechanisms and therapeutic implications. Pharmacol Ther. 2018;182:80–94. 28834698 10.1016/j.pharmthera.2017.08.009

[17] Silva VR, Santos LdS, Dias RB, Quadros CA, Bezerra DP. Emerging agents that target signaling pathways to eradicate colorectal cancer stem cells. Cancer Commun (Lond). 2021;41(12):1275–313. 34791817 10.1002/cac2.12235 PMC8696218

[18] Dejmek J, Dejmek A, Safholm A, Sjolander A, Andersson T. Wnt-5a protein expression in primary dukes B colon cancers identifies a subgroup of patients with good prognosis. Cancer Res. 2005;65(20):9142–6. 16230369 10.1158/0008-5472.CAN-05-1710

[19] Mikels AJ, Nusse R. Purified Wnt5a protein activates or inhibits beta-catenin-TCF signaling depending on receptor context. PLoS Biol. 2006;4(4):e115. 16602827 10.1371/journal.pbio.0040115 PMC1420652

[20] Beermann J, Piccoli MT, Viereck J, Thum T. Non-coding RNAs in Development and Disease: background, Mechanisms, and therapeutic approaches. Physiol Rev. 2016;96(4):1297–325. 27535639 10.1152/physrev.00041.2015

[21] Chen LL. The expanding regulatory mechanisms and cellular functions of circular RNAs. Nat Rev Mol Cell Biol. 2020;21(8):475–90. 32366901 10.1038/s41580-020-0243-y

[22] Wu Q, Liu W, Wang J, Zhu L, Wang Z, Peng Y. Exosomal noncoding RNAs in colorectal cancer. Cancer Lett. 2020;493:228–35. 32898600 10.1016/j.canlet.2020.08.037

[23] Ju H, Hu Z, Wei D, et al. A novel intronic circular RNA, circGNG7, inhibits head and neck squamous cell carcinoma progression by blocking the phosphorylation of heat shock protein 27 at Ser78 and Ser82. Cancer Commun (Lond). 2021;41(11):1152–72. 34498800 10.1002/cac2.12213 PMC8626595

[24] Patop IL, Wust S, Kadener S. Past, present, and future of circRNAs. EMBO J. 2019;38(16):e100836. 31343080 10.15252/embj.2018100836 PMC6694216

[25] Shan C, Zhang Y, Hao X, Gao J, Chen X, Wang K. Biogenesis, functions and clinical significance of circRNAs in gastric cancer. Mol Cancer. 2019;18(1):136. 31519189 10.1186/s12943-019-1069-0 PMC6743094

[26] Yang L, Wilusz JE, Chen LL. Biogenesis and Regulatory Roles of Circular RNAs. Annu Rev Cell Dev Biol. 2022;38:263–89. 35609906 10.1146/annurev-cellbio-120420-125117 PMC10119891

[27] Xiao MS, Ai Y, Wilusz JE. Biogenesis and Functions of circular RNAs come into Focus. Trends Cell Biol. 2020;30(3):226–40. 31973951 10.1016/j.tcb.2019.12.004 PMC7069689

[28] Zheng X, Huang M, Xing L, et al. The circRNA circSEPT9 mediated by E2F1 and EIF4A3 facilitates the carcinogenesis and development of triple-negative breast cancer. Mol Cancer. 2020;19(1):73. 32264877 10.1186/s12943-020-01183-9 PMC7137343

[29] An M, Zheng H, Huang J, et al. Aberrant Nuclear Export of circNCOR1 underlies SMAD7-Mediated lymph node metastasis of bladder Cancer. Cancer Res. 2022;82(12):2239–53. 35395674 10.1158/0008-5472.CAN-21-4349 PMC9359746

[30] Shen H, Liu B, Xu J, et al. Circular RNAs: characteristics, biogenesis, mechanisms and functions in liver cancer. J Hematol Oncol. 2021;14(1):134. 34461958 10.1186/s13045-021-01145-8 PMC8407006

[31] Chao F, Song Z, Wang S, et al. Novel circular RNA circSOBP governs amoeboid migration through the regulation of the miR-141-3p/MYPT1/p-MLC2 axis in prostate cancer. Clin Transl Med. 2021;11(3):e360. 33784000 10.1002/ctm2.360 PMC8002909

[32] Zhou M, Xiao MS, Li Z, Huang C. New progresses of circular RNA biology: from nuclear export to degradation. RNA Biol. 2021;18(10):1365–73. 33241761 10.1080/15476286.2020.1853977 PMC8489929

[33] Kong Y, Li Y, Luo Y, et al. circNFIB1 inhibits lymphangiogenesis and lymphatic metastasis via the miR-486-5p/PIK3R1/VEGF-C axis in pancreatic cancer. Mol Cancer. 2020;19(1):82. 32366257 10.1186/s12943-020-01205-6 PMC7197141

[34] Kristensen LS, Andersen MS, Stagsted LVW, Ebbesen KK, Hansen TB, Kjems J. The biogenesis, biology and characterization of circular RNAs. Nat Rev Genet. 2019;20(11):675–91. 31395983 10.1038/s41576-019-0158-7

[35] Dong H, Lei J, Ding L, Wen Y, Ju H, Zhang X. MicroRNA: function, detection, and bioanalysis. Chem Rev. 2013;113(8):6207–33. 23697835 10.1021/cr300362f

[36] Lee YS, Dutta A. MicroRNAs in cancer. Annu Rev Pathol. 2009;4:199–227. 18817506 10.1146/annurev.pathol.4.110807.092222 PMC2769253

[37] McDonald SL, Silver A. The opposing roles of Wnt-5a in cancer. Br J Cancer. 2009;101(2):209–14. 19603030 10.1038/sj.bjc.6605174 PMC2720208

[38] Wu G, Weng W, Xia P, et al. Wnt signalling pathway in bladder cancer. Cell Signal. 2021;79:109886. 33340660 10.1016/j.cellsig.2020.109886

[39] De A. Wnt/Ca2 + signaling pathway: a brief overview. Acta Biochim Biophys Sin (Shanghai). 2011;43(10):745–56. 21903638 10.1093/abbs/gmr079

[40] Hedgepeth CM, Conrad LJ, Zhang J, Huang HC, Lee VM, Klein PS. Activation of the wnt signaling pathway: a molecular mechanism for lithium action. Dev Biol. 1997;185(1):82–91. 9169052 10.1006/dbio.1997.8552

[41] Samatov TR, Tonevitsky AG, Schumacher U. Epithelial-mesenchymal transition: focus on metastatic cascade, alternative splicing, non-coding RNAs and modulating compounds. Mol Cancer. 2013;12(1):107. 24053443 10.1186/1476-4598-12-107 PMC3848796

[42] Li J, Sun D, Pu W, Wang J, Peng Y. Circular RNAs in Cancer: Biogenesis, function, and clinical significance. Trends Cancer. 2020;6(4):319–36. 32209446 10.1016/j.trecan.2020.01.012

[43] Conn SJ, Pillman KA, Toubia J, et al. The RNA binding protein quaking regulates formation of circRNAs. Cell. 2015;160(6):1125–34. 25768908 10.1016/j.cell.2015.02.014

[44] Jiang Y, Zhao J, Liu Y, et al. CircKPNB1 mediates a positive feedback loop and promotes the malignant phenotypes of GSCs via TNF-alpha/NF-kappaB signaling. Cell Death Dis. 2022;13(8):697. 35945192 10.1038/s41419-022-05149-1 PMC9363451

[45] Niu H, Zhang L, Wang B, et al. CircTUBD1 regulates Radiation-induced liver fibrosis response via a circTUBD1/micro-203a-3p/Smad3 positive feedback Loop. J Clin Transl Hepatol. 2022;10(4):680–91. 36062271 10.14218/JCTH.2021.00511 PMC9396324

[46] McConkey DJ, Choi W, Ochoa A, Dinney CPN. Intrinsic subtypes and bladder cancer metastasis. Asian J Urol. 2016;3(4):260–7. 29264194 10.1016/j.ajur.2016.09.009 PMC5730866

[47] Morra F, Merolla F, Criscuolo D, et al. CCDC6 and USP7 expression levels suggest novel treatment options in high-grade urothelial bladder cancer. J Exp Clin Cancer Res. 2019;38(1):90. 30786932 10.1186/s13046-019-1087-1 PMC6381716

[48] Lobo N, Mount C, Omar K, Nair R, Thurairaja R, Khan MS. Landmarks in the treatment of muscle-invasive bladder cancer. Nat Rev Urol. 2017;14(9):565–74. 28675174 10.1038/nrurol.2017.82

[49] Kamat AM, Hegarty PK, Gee JR, et al. ICUD-EAU International Consultation on bladder Cancer 2012: screening, diagnosis, and molecular markers. Eur Urol. 2013;63(1):4–15. 23083902 10.1016/j.eururo.2012.09.057

[50] Zhou WY, Cai ZR, Liu J, Wang DS, Ju HQ, Xu RH. Circular RNA: metabolism, functions and interactions with proteins. Mol Cancer. 2020;19(1):172. 33317550 10.1186/s12943-020-01286-3 PMC7734744

[51] Xu Y, Kang P, Leng K, et al. Circ_ASPH promotes cholangiocarcinoma growth and metastasis through the miR-581/ATP-binding cassette transporter G1 signaling pathway. Cancer Commun (Lond). 2020;40(10):545–50. 32735059 10.1002/cac2.12083 PMC7571393

[52] Yang Y, Luo D, Shao Y, et al. circCAPRIN1 interacts with STAT2 to promote tumor progression and lipid synthesis via upregulating ACC1 expression in colorectal cancer. Cancer Commun (Lond). 2023;43(1):100–22. 36328987 10.1002/cac2.12380 PMC9859733

[53] Meng S, Zhou H, Feng Z, et al. CircRNA: functions and properties of a novel potential biomarker for cancer. Mol Cancer. 2017;16(1):94. 28535767 10.1186/s12943-017-0663-2 PMC5440908

[54] Rybak-Wolf A, Stottmeister C, Glazar P, et al. Circular RNAs in the mammalian brain are highly abundant, conserved, and dynamically expressed. Mol Cell. 2015;58(5):870–85. 25921068 10.1016/j.molcel.2015.03.027

[55] Wan L, Zhang L, Fan K, Cheng ZX, Sun QC, Wang JJ. Circular RNA-ITCH suppresses Lung Cancer Proliferation via inhibiting the Wnt/beta-Catenin pathway. Biomed Res Int. 2016;2016:1579490. 27642589 10.1155/2016/1579490 PMC5013215

[56] Qin M, Liu G, Huo X, et al. Hsa_circ_0001649: a circular RNA and potential novel biomarker for hepatocellular carcinoma. Cancer Biomark. 2016;16(1):161–9. 26600397 10.3233/CBM-150552 PMC13016540

[57] Shang X, Li G, Liu H, et al. Comprehensive circular RNA profiling reveals that hsa_circ_0005075, a New Circular RNA biomarker, is involved in Hepatocellular Crcinoma Development. Med (Baltim). 2016;95(22):e3811. 10.1097/MD.0000000000003811 PMC4900729 27258521

