# Correction: ZEB1-mediated biogenesis of circnipbl sustains the metastasis of bladder cancer via Wnt/β-catenin pathway

**DOI:** 10.1186/s13046-025-03615-0

**Published:** 2026-02-06

**Authors:** Yao Kong, Yuanlong Li, Mingjie An, Yuming Luo, Hanhao Zheng, Yan Lin, Jiancheng Chen, Jin Yang, Libo Liu, Baoming Luo, Jian Huang, Tianxin Lin, Changhao Chan

**Affiliations:** 1 Department of Urology, Sun Yat-sen Memorial Hospital, Guangzhou, Guangdong P. R. China; 2 Guangdong Provincial Key Laboratory of Malignant Tumor Epigenetics and Gene Regulation, Sun Yat-sen Memorial Hospital, State Key Laboratory of Oncology in South China, Guangdong, P. R. China; 3 Department of Pancreatic Surgery, Department of General Surgery, Guangdong Provincial People’s Hospital, Guangdong Academy of Medical Sciences, Guangzhou, Guangdong P. R. China; 4 Department of Urology, Affiliated Hospital of Chengdu University, Chengdu, Sichuan P. R. China; 5 Department of Ultrasound, Sun Yat-sen Memorial Hospital, Guangzhou, Guangdong P. R. China

**Correction: J Exp Clin Cancer Res 42, 191 (2023).**

**https://doi.org/10.1186/s13046-023-02757-3**

Following the publication of the original article [1], the authors identified an unintentionally misused picture in the Fig. 3 caused by incaution of composed type.

The correct figure is presented below:

**Incorrect Fig. 3**

**Fig. 3:**
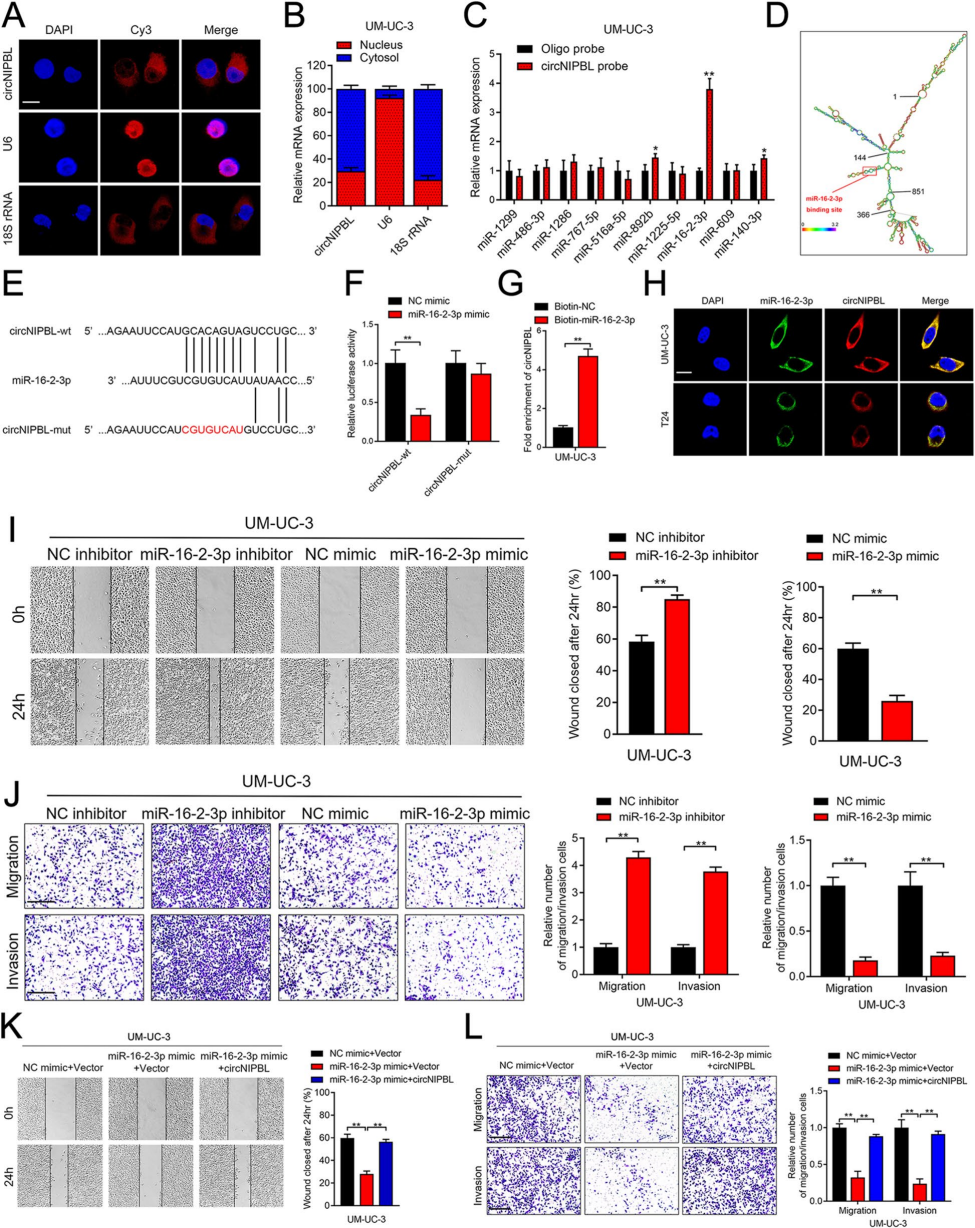
circNIPBL serves as a sponge for miR-16-2-3p in BCa cells.** A** FISH assay was used to detect the cellular localization of circNIPBL. Scale bar = 5 μm. **B** Subcellular fractionation assay was used to confirm the cellular localization of circNIPBL in UM-UC-3 cells. U6 was used for the nuclear control and 18S rRNA was used for the cytoplasmic control. **C** The expression level of ten predicted target miRNAs of circNIPBL were analyzed by qRT-PCR in UM-UC-3 cells. **D** RNAalifold was used to predict the secondary structure of circNIPBL. **E** Schematic illustrating the sequence alignment of circNIPBL with miR-16-2-3p. **F** Dual luciferase reporter assays showed that the luciferase activities of the circNIPBL-wt plasmid or circNIPBL-mut plasmid quantified following co-transfection with either the miR-16-2-3p or control mimics. **G** qRT-PCR analysis showed the circNIPBL captured by biotinylated miR-16-2-3p. **H** The co-localization of circNIPBL and miR-16-2-3p was detected by FISH assay. Scale bar = 5 μm. **I** Representative images and quantification of Wound healing assay showed the migration capability of UM-UC-3 cells transfected with miR-16-2-3p mimics or inhibitors. Scale bar = 100 μm. **J** Representative images and quantification of Transwell migration and Matrigel invasion assays showed the effect of UM-UC-3 cells transfected with miR-16-2-3p mimics or inhibitors. Scale bar = 100 μm. **K**,** L** Representative images and quantification of wound healing assay (K), Transwell migration and Matrigel invasion assays (L) by indicated UM-UC-3 cells. Scale bar = 100 μm. The statistical difference was assessed with one-way ANOVA followed by Dunnett tests in K and L; and the two-tailed Student *t* test in C, F, G, I and J; and the *χ*^*2*^ test in B. Error bars show the SD from three independent experiments. *p < 0.05 and **p < 0.01

**Correct Fig. 3**

**Fig. 3:**
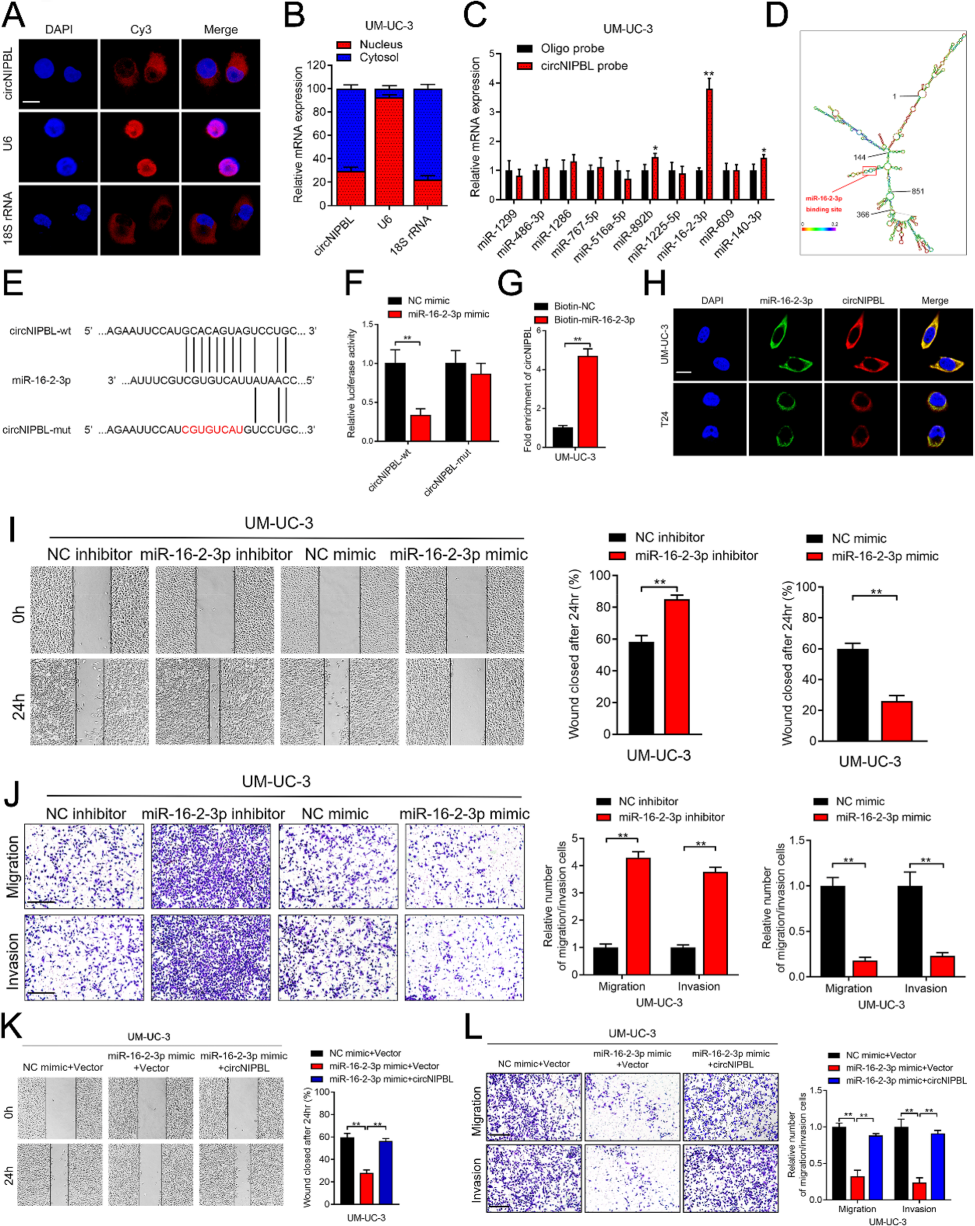
circNIPBL serves as a sponge for miR-16-2-3p in BCa cells. A FISH assay was used to detect the cellular localization of circNIPBL. Scale bar = 5 μm. B Subcellular fractionation assay was used to confirm the cellular localization of circNIPBL in UM-UC-3 cells. U6 was used for the nuclear control and 18 S rRNA was used for the cytoplasmic control. C The expression level of ten predicted target miRNAs of circNIPBL were analyzed by qRT-PCR in UM-UC-3 cells. D RNAalifold was used to predict the secondary structure of circNIPBL. E Schematic illustrating the sequence alignment of circNIPBL with miR-16-2-3p. F Dual luciferase reporter assays showed that the luciferase activities of the circNIPBL-wt plasmid or circNIPBL-mut plasmid quantified following co-transfection with either the miR-16-2-3p or control mimics. G qRT-PCR analysis showed the circNIPBL captured by biotinylated miR-16-2-3p. H The co-localization of circNIPBL and miR-16-2-3p was detected by FISH assay. Scale bar = 5 μm. I Representative images and quantification of Wound healing assay showed the migration capability of UM-UC-3 cells transfected with miR-16-2-3p mimics or inhibitors. Scale bar = 100 μm. J Representative images and quantification of Transwell migration and Matrigel invasion assays showed the effect of UM-UC-3 cells transfected with miR-16-2-3p mimics or inhibitors. Scale bar = 100 μm. K, L Representative images and quantification of wound healing assay (K), Transwell migration and Matrigel invasion assays (L) by indicated UM-UC-3 cells. Scale bar = 100 μm. The statistical difference was assessed with one-way ANOVA followed by Dunnett tests in K and L; and the two-tailed Student t test in C, F, G, I and J; and the χ2 test in B. Error bars show the SD from three independent experiments. *p < 0.05 and **p < 0.01

After carefully checking the original data, we confirmed that we mistakenly selected the images during the process of image label and storage. Since the error does not originate from original experiments, this correction does not affect the overall conclusion of this article. The original article has been corrected.
