# Supplementary material for: ZEB1-mediated biogenesis of circNIPBL sustains the metastasis of bladder cancer via Wnt/β-catenin pathway
Source: J Exp Clin Cancer Res. 2023 Aug 2;42:191. doi: 10.1186/s13046-023-02757-3 (PMC10394821; doi:10.1186/s13046-023-02757-3)
Supplement: Supplementary file 1 — Supplementary Material 1 [file 13046_2023_2757_MOESM1_ESM.pdf]

AJE®

Editing Certificate

This document certifies that the manuscript

ZEB1 mediated circNIPBL biogenesis sustains bladder cancer metastasis via a miR-16-2-3p/Wnt5a Positive Feedback Loop

prepared by the authors

Yuanlong Li1,2#, Yao Kong3#, Mingjie An1,2#, Yuming Luo3, Hanhao Zheng1,2, Yan Lin1,2, Jiancheng Chen1,2, Jin Yang4, Libo Liu1,2, Baoming Luo5, Jian Huang1,2*, Tianxin Lin1,2*, Changhao Chen1,2*

was edited for proper English language, grammar, punctuation, spelling, and overall style by one or more of the highly qualified native English speaking editors at AJE.

This certificate was issued on **April 3, 2023** and may be verified on the AJE website using the verification code **4E5E-7DEB-AA1D-7BF3-ACAP**.

AJE® EDITING CERTIFIED

Neither the research content nor the authors' intentions were altered in any way during the editing process. Documents receiving this certification should be English-ready for publication; however, the author has the ability to accept or reject our suggestions and changes. To verify the final AJE edited version, please visit our verification page at aje.com/certificate. If you have any questions or concerns about this edited document, please contact AJE at support@aje.com.

AJE provides a range of editing, translation, and manuscript services for researchers and publishers around the world.
For more information about our company, services, and partner discounts, please visit aje.com.
