# Supplementary material for: ZEB1-mediated biogenesis of circNIPBL sustains the metastasis of bladder cancer via Wnt/β-catenin pathway
Source: J Exp Clin Cancer Res. 2023 Aug 2;42:191. doi: 10.1186/s13046-023-02757-3 (PMC10394821; doi:10.1186/s13046-023-02757-3)
Supplement: Supplementary file 2 — Supplementary Material 2 [file 13046_2023_2757_MOESM2_ESM.docx]

**Supplementary data**

**
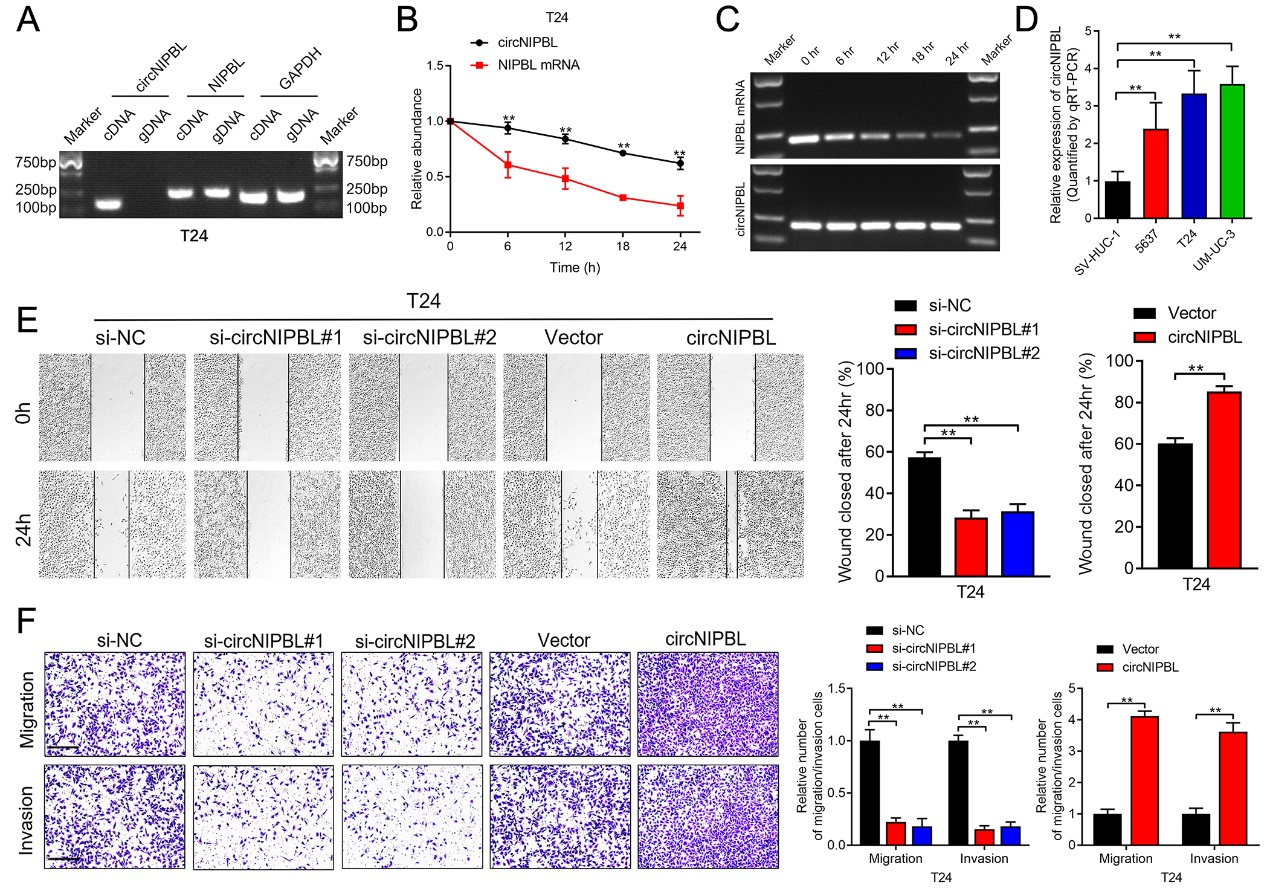
Fig. S1** **circNIPBL promotes the proliferation, migration, and invasion of BCa cells *in vitro*. A** PCR analysis for circNIPBL and NIPBL in the cDNA and gDNA of T24 cells. GAPDH was used as normal control. **B, C** Actinomycin D assay (B) and agarose gel electrophoresis assay (C) to assess the stability of circNIPBL and NIPBL mRNA in T24 cells at the indicated time points. **D** Relative expression of circNIPBL in UM-UC-3, T24, 5637 and SV-HUC-1 cell lines was detected using qRT-PCR. **E** Representative images and quantification of Wound healing assay of T24 cells treated with circNIPBL-downregulated or circNIPBL-overexpressing. Scale bar=100μm. **F** Representative images and quantification of Transwell migration and Matrigel invasion assays of T24 cells treated with circNIPBL-downregulated or -overexpressing. Scale bar=100μm. The statistical difference was assessed with one-way ANOVA followed by Dunnett tests in D-F; and the two-tailed Student *t* test in B, E and F. Error bars show the SD from three independent experiments. **p* < 0.05 and ***p* < 0.01.


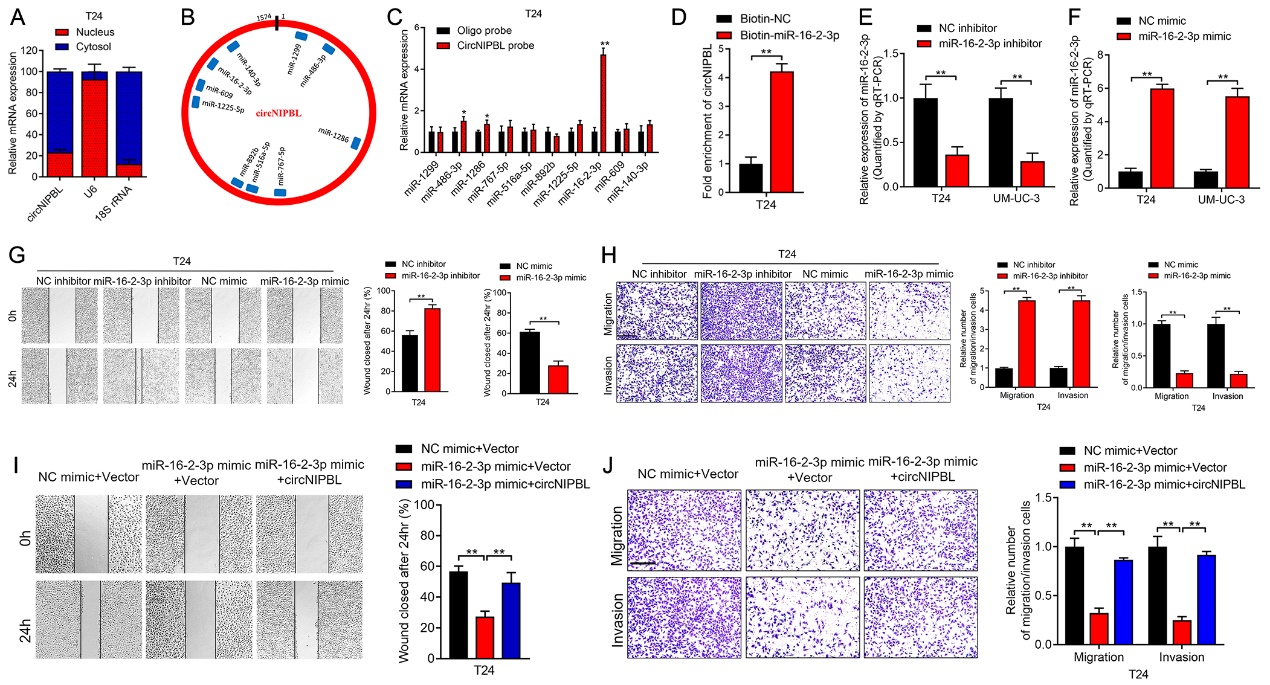


**Fig. S2** **miR-16-2-3p inhabits the proliferation, migration, and invasion of BCa cells *in vitro*.** **A** Subcellular fractionation assay was used to confirm the cellular localization of circNIPBL in T24 cells. U6 was used for the nuclear control and 18S rRNA was used for the cytoplasmic control. **B** RegRNA 2.0 was used to predict the potential target miRNAs of circNIPBL. **C** The expression level of ten predicted target miRNAs of circNIPBL were analyzed by qRT-PCR in T24 cells. **D** qRT-PCR analysis of the circNIPBL captured by biotinylated miR-16-2-3p. **E, F** qRT-PCR was used to assess the expression level of miR-16-2-3p in miR-16-2-3p inhibitors (E), mimics (F), and paired control BCa cells. **G** Representative images and quantification of Wound healing assay of T24 cells treated with miR-16-2-3p mimics or inhibitors. Scale bar=100μm. **H** Representative images and quantification of Transwell migration and Matrigel invasion assays of T24 cells treated with miR-16-2-3p mimics or inhibitors. Scale bar=100μm. **I, J** Representative images and quantification of wound healing assay (I), Transwell migration and Matrigel invasion assays (J) in indicated T24 cells. Scale bar=100μm. The statistical difference was assessed with one-way ANOVA followed by Dunnett tests in I and J; and the two-tailed Student *t* test in C-H; and the *χ^2^* test in A. Error bars show the SD from three independent experiments. **p* < 0.05 and ***p* < 0.01.

**
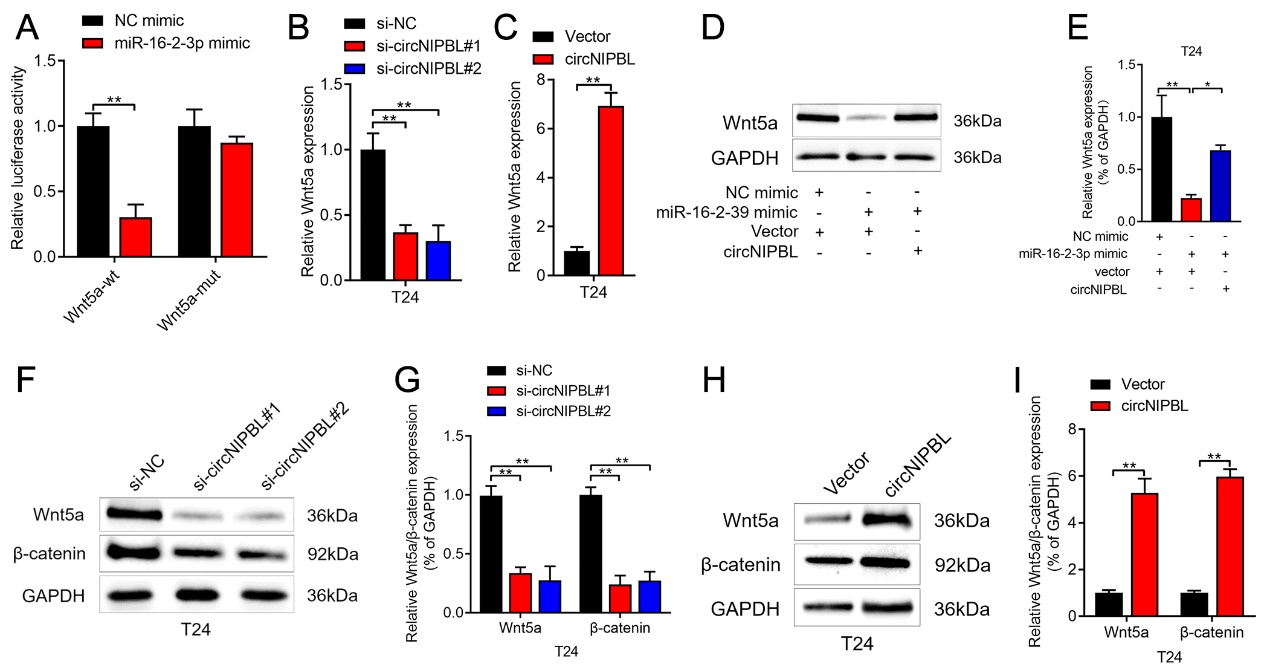
Fig. S3 circNIPBL attenuates miR-16-2-3p-mediated Wnt signaling suppression. A** The luciferase activities of the Wnt5a-wt plasmid or Wnt5a-mut plasmid quantified following transfecting control mimic or miR-16-2-3p mimic into T24 cells. **B, C** qRT-PCR analysis of the impact of circNIPBL knockdown (B) or circNIPBL overexpression (C) on Wnt5a expression in T24 cells. **D, E** Representative images (D) and quantification (E) of Western blotting assay of Wnt5a in indicated in T24 cells. **F-I** Western blotting assay showed the expression levels of Wnt5a and β-catenin after circNIPBL knockdown (F, G) or circNIPBL overexpression (H, I) in T24 cells. The statistical difference was assessed with one-way ANOVA followed by Dunnett tests in B, E and G; and the two-tailed Student *t* test in A, C and I. Error bars show the SD from three independent experiments. **p* < 0.05 and ***p* < 0.01.


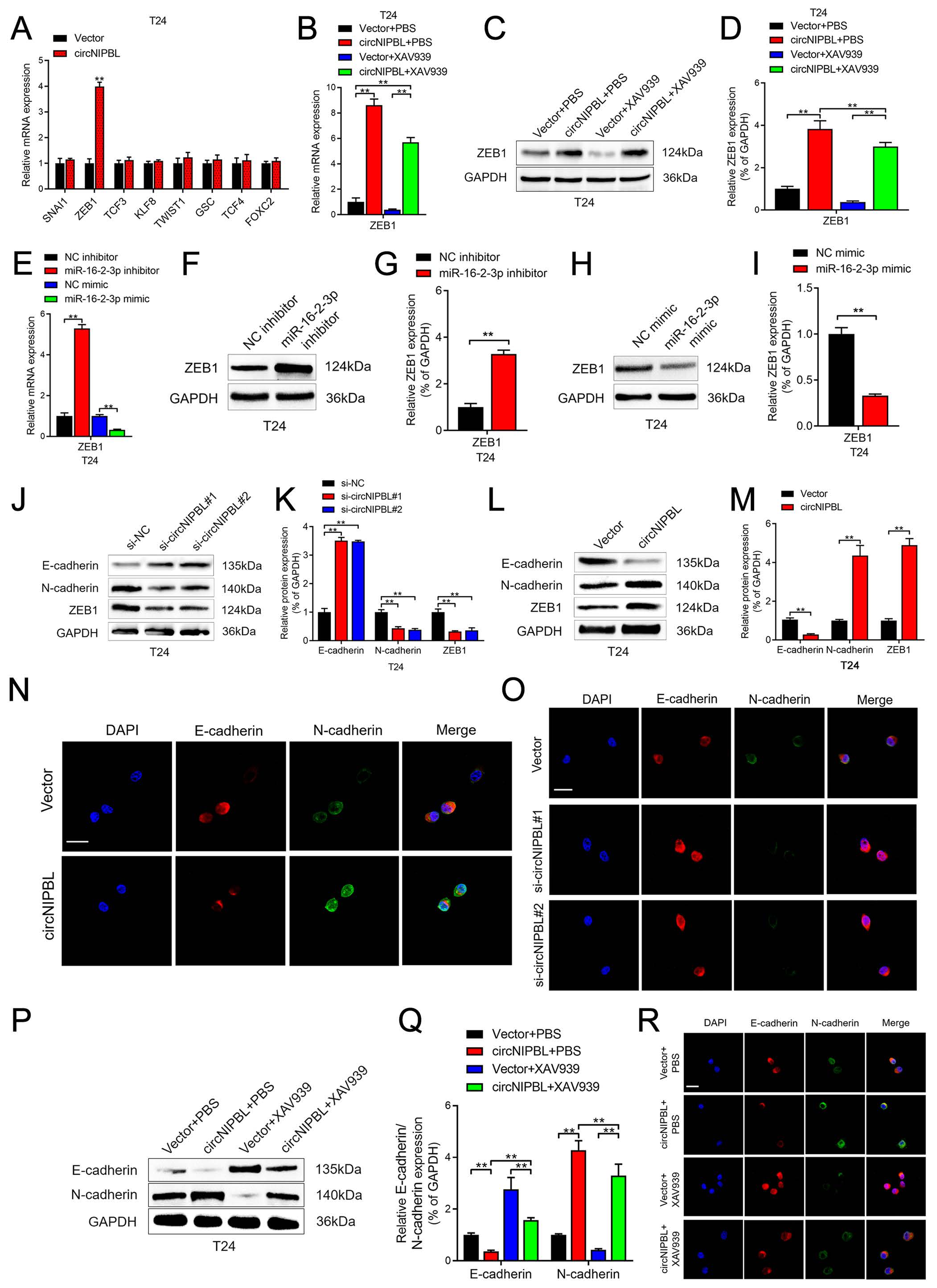
**Fig. S4** **circNIPBL promotes EMT via activating miR-16-2-3p/Wnt5A/ZEB1 axis in BCa. A** qRT-PCR analysis showed that the expressions of downstream targets of Wnt signaling pathway in circNIPBL overexpressing T24 cells. **B** qRT-PCR analysis of ZEB1 expression in indicated T24 cells. **C, D** Representative images (C) and quantification (D) of Western blotting assay of ZEB1 in indicated T24 cells. **E** qRT-PCR analysis of ZEB1 expression in indicated T24 cells. **F-I** Representative images and quantification of Western blotting assay of ZEB1 in T24 cells transfected with miR-16-2-3p inhibitors (F, G) and mimics (H, I). **J-M** Representative images and quantification of Western blotting assay of N-cadherin, E-cadherin, ZEB1 after circNIPBL knockdown (J, K) or circNIPBL overexpression (L, M) in T24 cells. **N, O** The expression of N-cadherin and E-cadherin was detected by IF assay in circNIPBL knockdown (N) or circNIPBL overexpression (O) T24 cells. Scale bar=5μm. **P, Q** Representative images (P) and quantification (Q) of Western blotting assay of N-cadherin and E-cadherin in indicated T24 cells. **R** The expression of N-cadherin and E-cadherin was detected by IF assay in indicated T24 cells. Scale bar=5μm. The statistical difference was assessed with one-way ANOVA followed by Dunnett tests in B, D, E, K and R; and the two-tailed Student *t* test in A, G, I and M. Error bars show the SD from three independent experiments. **p* < 0.05 and ***p* < 0.01.


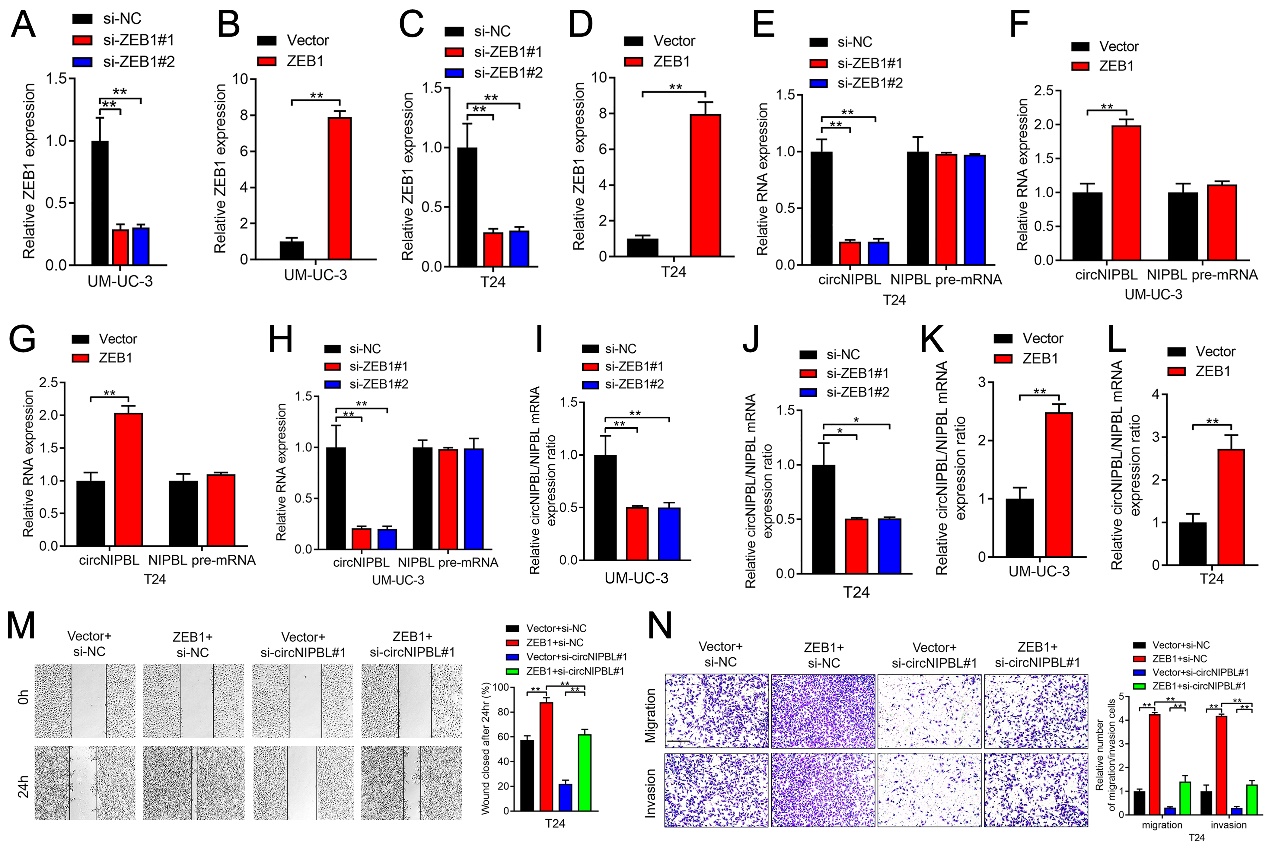


**Fig. S5 ZEB1-mediated circNIPBL biogenesis sustains the EMT activation of BCa. A-D** qRT-PCR analysis showed that the expression level of ZEB1 in ZEB1 knockdown (A, C) and overexpression (B, D) BCa cells. **E-H** qRT-PCR analysis showed that the expression level of circNIPBL and NIPBL pre-mRNA in ZEB1 knockdown (E, H), ZEB1 overexpression (F, G), and paired control BCa cells. **I-L** qRT-PCR analysis showed that the circNIPBL and NIPBL mRNA expression ratio in ZEB1 knockdown (I, J), ZEB1 overexpression (K, L) BCa cells. **M, N** Representative images and quantification of Wound healing (M) and Transwell migration and Matrigel invasion (N) assays in indicated T24 cells. Scale bar=100μm. The statistical difference was assessed with one-way ANOVA followed by Dunnett tests in A, C, E, H, I, J, M and N; and the two-tailed Student *t* test in B, D, F, G, K and L. Error bars show the SD from three independent experiments. **p* < 0.05 and ***p* < 0.01.


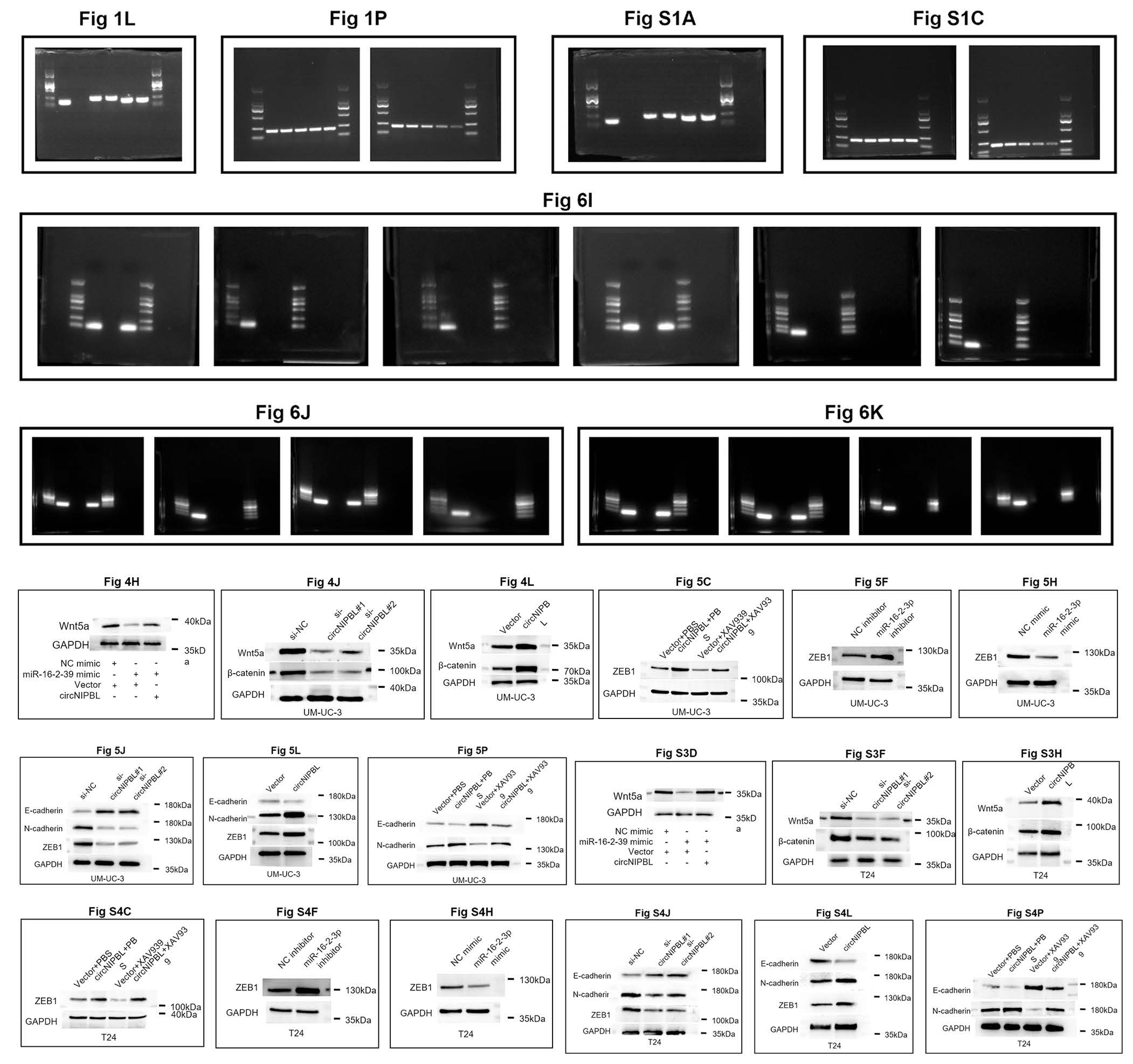


**Fig. S6 Full uncut original pictures.**

**Table S1. Univariate and multivariate analysis of Overall Survival (OS) for circNIPBL expression in BCa patients (*n* = 296)**

| **Variables** | | **Univariate analysis** | | | | | | **Multivariate analysis** | | | |
| --- | --- | --- | --- | --- | --- | --- | --- | --- | --- | --- | --- |
|  |  | **HR** | | **95%CI** | | ***P*-value^ⅰ^** | | **HR** | **95%CI** | | ***P*-value^ⅰ^** |
| Age (<65 vs. ≥65) | 1.367 | | 0.959-1.946 | | 0.083 | |  | |  |  | |
| Gender (Male vs. Female) | 0.890 | | 0.613-1.293 | | 0.542 | |  | |  |  | |
| Grade (High vs. Low) | 1.096 | | 0.758-1.586 | | 0.625 | |  | |  |  | |
| Lymphatic metastasis  (positive vs. negative) | 1.896 | | 1.327-2.709 | | **0.001^**^** | | 1.891 | | 1.323-2.703 | **0.001^*^** | |
| circNIPBL expression  (High vs. Low) | 1.592 | | 1.124-2.256 | | **0.009^**^** | | 1.588 | | 1.120-2.252 | **0.009^**^** | |

Abbreviations: HR = hazard ratio; 95% CI = 95% confidence interval; T grade = tumor grade. ^i^Cox regression analysis, ^*^*p* <0.05, ^**^*p* <0.01.

**Table S2. Univariate and multivariate analysis of Disease-Free Survival (DFS) for circNIPBL expression in BCa patients (*n* = 296)**

| **Variables** | | **Univariate analysis** | | | | | | **Multivariate analysis** | | | |
| --- | --- | --- | --- | --- | --- | --- | --- | --- | --- | --- | --- |
|  |  | **HR** | | **95%CI** | | ***P*-value^ⅰ^** | | **HR** | **95%CI** | | ***P*-value^ⅰ^** |
| Age (<65 vs. ≥65) | 0.466 | | 0.823-1.529 | | 0.466 | |  | |  |  | |
| Gender (Male vs. Female) | 1.134 | | 0.809-1.590 | | 0.465 | |  | |  |  | |
| Grade (High vs. Low) | 0.972 | | 0.710-1.332 | | 0.861 | |  | |  |  | |
| Lymphatic metastasis  (positive vs. negative) | 1.578 | | 1.152-2.163 | | **0.005^**^** | | 1.576 | | 1.149-2.162 | **0.005^*^** | |
| circNIPBL expression  (High vs. Low) | 1.705 | | 1.261-2.306 | | **0.001^**^** | | 1.704 | | 1.260-2.306 | **0.001^**^** | |

Abbreviations: HR = hazard ratio; 95% CI = 95% confidence interval; T grade = tumor grade. ^i^Cox regression analysis, ^*^*p* <0.05, ^**^*p* <0.01.

**Table S3. Primer and probes used in the experiments.**

| **Gene** | | **Sequence (5’-3’)** | **Application** |
| --- | --- | --- | --- |
| circNIPBL | F: GCTGAAATTGAGCGAATAGAGAG  R: GGCATATCCCCATTCATCCTG | | qRT-PCR |
| NIPBL | F: CGATTTGTCTTCTCGGCTGG  R: AGGTGCCAGCTGTCATTACT | | qRT-PCR |
| GAPDH | F: CACATCGCTCAGACACCATG  R: TGACGGTGCCATGGAATTTG | | qRT-PCR |
| U1 | F: CAGGGGAGATAACGTGACCA  R: GGGAAAAGCACGGACACAG | | qRT-PCR |
| 18S rRNA | F: AGCAGACATTGACCTCACCA  R: CCTCTATGGGCCCGAATCTT | | qRT-PCR |
| miR-16-2-3p | F: GCCCAATATTACTGTGCTG | | qRT-PCR |
| miR-1299 | F: UUCUGGAAUUCUGUGUGAGGGA | | qRT-PCR |
| miR-486-3p | F: UCCUGUACUGAGCUGCCCCGAG | | qRT-PCR |
| miR-1286 | F: UGCAGGACCAAGAUGAGCCCU | | qRT-PCR |
| miR-767-5p | F: UGCACCAUGGUUGUCUGAGCAUG | | qRT-PCR |
| miR-516a-5p | F: UUCUCGAGGAAAGAAGCACUUUC | | qRT-PCR |
| miR-892b | F: CACUGGCUCCUUUCUGGGUAGA | | qRT-PCR |
| miR-1225-5p | F: GUGGGUACGGCCCAGUGGGGGG | | qRT-PCR |
| miR-609 | F: AGGGUGUUUCUCUCAUCUCU | | qRT-PCR |
| miR-140-3p | F: UACCACAGGGUAGAACCACGG | | qRT-PCR |
| Wnt5a | F: CGTTAGCAGCATCAGTCCAC  R: ACGGCATCTCTCTTTCACCA | | qRT-PCR |
| Wnt9a | F: CCTCTATGCCATCTCCTCGG  R: TTCCTTGACGAACTTGCTGC | | qRT-PCR |
| Wnt3a | F: GGACAAAGCTACCAGGGAGT  R: ACCATCCCACCAAACTCGAT | | qRT-PCR |
| FZD5 | F: TAGCGGTTTTGTGTTCAGCC  R: TGCCATCTCACCAGCCTAAA | | qRT-PCR |
| RNF43 | F: AGTGGGGAGACTAGCACCTA  R: ATGAATCGGAGCCTAGCCTC | | qRT-PCR |
| LRP5 | F: CGCCAAGACAGACAAGATCG  R: CTTGGCCACATTCACAGCTT | | qRT-PCR |
| LRP6 | F: ATCTCCGGCGAATTGAAAGC  R: TCTACCCTCTCGACCTGTCA | | qRT-PCR |
| ZNRF3 | F: TCTGAAGACCCGCTCAAGAG  R: GAGACCACGACGAAGAAAGC | | qRT-PCR |
| SNAI1 | F: CTAGAGTCTGAGATGCCCCG  R: AGTTCTGGGAGACACATCGG | | qRT-PCR |
| TCF3 | F: ATGGGGCATTTTGTTGGGAC  R: TCCTGTCTACGTCACGATGG | | qRT-PCR |
| KLF8 | F: CGCAGCTTTTCTCGTTCTGA  R: GGCTGCTTCAACCCCATATG | | qRT-PCR |
| TWIST1 | F: TTCAAAGAAACAGGGCGTGG  R: GCACGACCTCTTGAGAATGC | | qRT-PCR |
| GSC | F: CAGAAGCGGTCCTCATCAGA  R: TCTGTGCAAGTCCTTCGAGT | | qRT-PCR |
| TCF4 | F: CTTCCTCCAAACCAGCAACC  R: ATGTGGATGCAGGCTACAGT | | qRT-PCR |
| FOXC2 | F: AAGGTGGTGATCAAGAGCGA  R: GGTCATGATGTTCTCCACGC | | qRT-PCR |
| ZEB1 | F: CTCTTTCAGCATCACCAGGC  R: CAGAACAACAGCTTGCACCA | | qRT-PCR |
| miR-16-2-3p  mimics | Sense: GCCCAATATTACTGTGCTG  Antisense: CAGCACAGTAATATTGGGC | | miRNA  mimics |
| miR-16-2-3p  inhibitor | CAGCACAGTAATATTGGGC | | miRNA  inhibitor |
| si-circNIPBL#1 | Sense: CAAGAUAAAGUGUUUGGGAAA  Antisense:UUUCCCAAACACUUUAUCUUG | | siRNA |
| si-circNIPBL#2 | Sense: AGAUAAAGUGUUUGGGAAAUG  Antisense:CAUUUCCCAAACACUUUAUCU | | siRNA |
| si-ZEB1#1 | Sense: GGUAGAUGGUAAUGUAAUATT  Antisense: UAUUACAUUACCAUCUACCTT | | siRNA |
| si-ZEB1#2 | Sense: GGUGUAAUCGUAAAUUCAATT  Antisense: UUGAAUUUACGAUUACACCTT | | siRNA |
|  |  | |  |

**Table S4. Antibodies used in the experiments.**

| **Product** | **Source** | **No. of Catalogue** |
| --- | --- | --- |
| **Primary antibody:** |  |  |
| **Western blot:** |  |  |
| anti-Wnt5a | Cell Signaling Technology | 2392S |
| anti-GAPDH | Abcam | ab8245 |
| anti-β-catenin | Cell Signaling Technology | 8480S |
| anti-E-cadherin | Cell Signaling Technology | 3195S |
| anti-N-cadherin | Cell Signaling Technology | 13116S |
| anti-ZEB1 | Cell Signaling Technology | 70512S |
| **Immunofluorescence:** |  |  |
| anti-ZEB1 | Cell Signaling Technology | 70512S |
| anti-E-cadherin | Cell Signaling Technology | 3195S |
| anti-N-cadherin | Cell Signaling Technology | 13116S |
| **Secondary antibody:** |  |  |
| **Western blot:** |  |  |
| anti-rabbit IgG-HRP | Cell Signaling Technology | 7074 |
| anti-mouse IgG-HRP | Cell Signaling Technology | 7076 |
| **Immunofluorescence:** |  |  |
| [Alexa Fluor 59](https://www.baidu.com/link?url=nF9d2Xaur7vyZuSh6bwYgXJHxCoqgi5ljmVkB6q--I4j4E8mmfQwWu1WHii3mT9LmMQ5XQE23xsmXGJKAUgtmctb7vuX9L1odcCwYmhMNRgtKDKfaFBBlPGw7dU_i5LpQii0iVI7_AAuccqxBIw54_&wd=&eqid=cad6a7aa0006a5040000000661054afd)4 | Invitrogen | A32773 |
| [Alexa Fluor 4](https://www.baidu.com/link?url=nF9d2Xaur7vyZuSh6bwYgXJHxCoqgi5ljmVkB6q--I4j4E8mmfQwWu1WHii3mT9LmMQ5XQE23xsmXGJKAUgtmctb7vuX9L1odcCwYmhMNRgtKDKfaFBBlPGw7dU_i5LpQii0iVI7_AAuccqxBIw54_&wd=&eqid=cad6a7aa0006a5040000000661054afd)88 | Invitrogen | A32766 |

### Supplemental methods

**RNA extraction and quantitative real-time PCR (qRT-PCR)**

Total RNAs were extracted from patient specimens or BCa cells using TRizol reagent (Invitrogen Life Technologies) following the manufactory’s instruction. Then, transcribing the total RNA into cDNA by PrimerScript RT Master Mix (Takara, Japan). The qRT-PCR assay was performed with TB Green Premix Ex Taq II (Takara, Japan) according to the manufacture’s instruction. The process was conducted on Light Cycler 480 Detection System (Roche) and GAPDH was used as the internal control. cDNA and qRT-PCR were performed with Mir-XTM miRNA First-Strand Synthesis Kit (Takara, Japan) for miRNA.

**Wound healing assay**

3×10^5^ BCa cells were added to each hole of 6-well plate to ensure that the cell density reach 100% the next day. Next, discarded the culture medium and scratched with 20 μl pipette tips to create a wound. Then the plate was placed in the incubator and the migration ability of BCa cells was measured by the distance of wound closure between two sides at 0h and 24 h time points.

**Transwell assay**

5×10^5^ BCa cells suspended with 200ul serum-free medium were added to the upper chamber of Matrigel-coated Transwell plates. 700ul medium containing 10% FBS was applied to the lower chamber of Transwell chambers (BD Biosciences, MA, USA). 12 hours later, the cells remaining in the upper chamber were scrapped off and the invaded cells were fixed with 4% paraformaldehyde, stained by crystal violet, and the number of migrated cells were captured and calculated in 4 random fields.

**Subcellular fractionation assay**

According to the manufactory’s instruction, the NE-PER™ Nuclear and Cytoplasmic Extraction Reagents were using to separate the cytoplasmic and nuclear fraction of BCa cells. Briefly, 1 x 10^6^ BCa cells were digested and re-suspended with PBS. Then CER I, CERII were added into the suspension. After incubating on ice for 10 min, the suspension was centrifuged at 16000 x g for 5 min and the supernatant with plasma portion was kept. The pellet was resuspended with ice-cold NER and vortexed 15 seconds every 10 min for four times. Then the suspension was centrifuged for 10 min at 16,000 x g and the supernatant with nuclear fraction was collected. The plasma portion and nuclear fraction was kept at -80°C until use.

**Fluorescence in situ hybridization (FISH)**

2×10^4^ BCa cells were added into the confocal dishes and fixed with 4% paraformaldehyde. Then we permeabilizated the cells with 0.5% Triton and washed the cells with PBS for 3 times. After prehybridization, the cells were hybridized with Fish probe at 37°C overnight, cell nuclei were stained with DAPI. The images were capture with Zeiss LAM 710 focal Microscope (Carl Zeiss AG, Germany).

**Immunofluorescence (IF) assay**

The indicated cells were seeded into confocal dishes one day in advance. Then, the cells were fixed with 4% paraformaldehyde for 15 min, after which the cells were treated with 0.5% Triton X-100 (Sigma-Aldrich) for 15 min at room temperature to permeabilize the cells. Subsequently, the cells were blocked with goat serum at 37° for 1 h, followed by the incubation with primary antibodies at 4°C overnight. After staining with the corresponding fluorescent secondary antibodies for 1 h at room temperature, the nuclei were stained with DAPI for another 10 min. The images were capture with Zeiss LAM 710 focal Microscope (Carl Zeiss AG, Germany).

**Colocalization of circNIPBL and miR-16-2-3p**

The colocalization of circNIPBL and miR-16-2-3p was confirmed by fluorescence in situ hybridization. Briefly, UM-UC-3 and T24 cells were seeded on a confocal plate and when reached 80-90% confluence, the cells were fixed with 4% [paraformaldehyde](javascript:;) and permeabilizated with 0.5% Triton. After washing with PBS for 3 times, the cells were hybridized with circNIPBL probe and miR-16-2-3p probe overnight at 37°C in 2×SSC, 10% formamide and 10% dextran. Cell nuclei were stained with DAPI. The images were capture with Zeiss LAM 710 focal Microscope (Carl Zeiss AG, Germany).

**Western blotting**

To determine the protein levels in cells, cells were washed with PBS for three times and treated with RIPA buffer (Invitrogen) mixed with 1% Protease Inhibitor Cocktail and 1% phosphatase inhibitor. After centrifugation at 12000g, 4℃ for 30 min, the supernatants were transferred and t protein concentration was measured. Then, proteins were separated by SDS-PAGE gels. Then proteins were transferred to PVDF membranes from SDS-PAGE gels. Then the membranes incubated with primary antibodies at 4°C overnight and further incubated with secondary antibody, the protein strips were washed with TBST and detected with ECL chemiluminescence kit (Pierce).

**Immunohistochemistry**

Paraffin-embedded samples were deparaffinized in xylene twice, and rehydrated in [absolute](javascript:;) [ethyl](javascript:;) [alcohol](javascript:;) twice, followed by grade series of ethanol. Then the samples were submerged in EDTA buffer solution and heated for 7 min with medium-high heat, 14 min with medium-low heat for epitope retrieval. The activity of endogenous peroxidase was blocked with 3% hydrogen peroxide at room temperature, for 10 min. Then the samples were blocked in goat serum for 20 min. Incubate the slice with primary antibody at 4℃ overnight and secondary antibody at room temperature for 2h. After counterstaining with hematoxylin, the immunohistochemistry analyses were scored by 2 independent professional pathologists. Images were visualized using a Nikon ECLIPSE Ti (Fukasawa, Japan) microscope system and processed with Nikon software.

**FISH Immunohistochemistry**

The RNA FISH kit (GenePharma Co., Ltd., Shanghai, China) was used in BCa tissues to evaluate the expression level of circNIPBL, E-cadherin and N-cadherin according to the manufacture instruction. The sections were digested with proteinase K and the cells were hybridized with Fish probe at 37°C overnight. The nucleus was stained with DAPI. The images were capture with Zeiss LAM 710 focal Microscope (Carl Zeiss AG, Germany).

**RNA immunoprecipitation (RIP)**

EZ-Magna RIP kit (Merck, Darmstadt, Germany, Cat#17-701) was used to confirm the binding sites of ZEB1 and NIPBL pre-mRNA. 2×10^7^ BCa cells were lysed by RIP lysis buffer at 4°C for 10 min and centrifugated for 10 min at 120,000 g. Subsequently, the cell extracts were co-immunoprecipitated with magnetic beads conjugated with antibody overnight. After eluting and purifying, the retrieved RNA was analyzed by qRT-PCR.

**Agarose gel electrophoresis**

1% agarose was prepared by mixing 0.8g agarose with 80ml 1×TAE buffer and heating them in the microwave oven to fully dissolve the agarose. Then 8ul Solargel Red (Solarbio) was added in the solution. When the gel is cooled to about 50℃, evenly pour it into the plastic tank until completely cooled. Put the plastic tank into the electrophoresis tank, and make sure it was completely soaked in TAE buffer. Mix DNA samples with sample buffer and added 20ul DNA sample to each sample hole. Connect the positive and negative electrophoresis tank, set the voltage at 100V for 30 min and started electrophoresis. The DNA bands were imaged under UV imaging system.

**RNase R treatment**

Total RNA from cells was extracted using the Trizol reagent (Takara, Shiga, Japan) and subsequently divided into two aliquots: one was used for RNase R digestion and 2μg of total RNA was mixed with buffer and incubated for 15min at 37°C in 3U/mg RNase R (Epicentre Technologies, Madison, WI, USA); the other aliquot was the control, which was treated with 0.2μl of DEPC water. GAPDH was used as the internal control.

**Actinomycin D assay**

1×10^5^ BCa cells were inoculated per well in a 6-well plate and incubated overnight. The cells were then treated with 2μg/ml actinomycin D (Sigma) at specified points in time (4, 8, 12 and 24 h). After extracting total RNA, qRT-PCR analyzed the expression of circNIPBL.

**Dual luciferase assay**

1×10^5^ BCa cells were seeded in 6-well plates to co-transfect the plasmids carrying wild-type circNIPBL with miR-16-2-3p mimics or NC mimic. Mutant sequences of circNIPBL were also co-transfected with miR-16-2-3p mimics or NC mimics. Then the transfected Cells were seeded into 96-well plates at a density of 1×10^4^ cells per well, the luciferase activities were measured according to the instruction of Dual-Luciferase Reporter Assay (Promega, USA).
